# Glutamine from plasma-like medium fuels nitrogen metabolism and influences proliferation in *Leptospira interrogans*

**DOI:** 10.1371/journal.ppat.1014534

**Published:** 2026-10-08

**Authors:** Matthew H. Ward, Nathan Scherer, Leah P. Shriver, Gary J. Patti

**Affiliations:** 1 Department of Chemistry, Washington University in St. Louis, St. Louis, Missouri, United States of America; 2 Department of Genetics, Washington University in St. Louis, St. Louis, Missouri, United States of America; 3 Department of Medicine, Washington University in St. Louis, St. Louis, Missouri, United States of America; 4 Center for Mass Spectrometry and Metabolic Tracing, United States of America; University of Nebraska Medical Center College of Medicine, UNITED STATES OF AMERICA

## Abstract

Leptospirosis, caused by pathogenic *Leptospira* spp. such as *L. interrogans*, is a bacterial zoonosis of increasing prevalence with no consistently effective treatments in severe cases. We sought to characterize metabolic mechanisms that support *L. interrogans* infection, with the ultimate goal of revealing unexplored therapeutic opportunities. We first established and validated a culture medium, which we refer to as supplemented Human Plasma-Like Medium (sHPLM). sHPLM more closely resembles the physiological environment of the human host than standard culture media, such as the EMJH (Ellinghausen-McCullough-Johnson-Harris) medium typically used for *Leptospira* culture. We also pioneered metabolomics approaches to study sHPLM-cultured *Leptospira*. Specifically, we developed a liquid chromatography/mass spectrometry (LC/MS) workflow for both medium metabolomics and stable isotope tracing with *L. interrogans* cultures. The application of these technologies in *L. interrogans* revealed that the amino acid glutamine is a major nitrogen source among the many available in sHPLM. Subsequent growth studies in three pathogenic *Leptospira* strains and the nonpathogenic strain *L. biflexa* Patoc-1 showed robust proliferation when only glutamine-derived nitrogen was available. Further, a small-molecule inhibitor designed to target glutamine utilization, JHU-083, effectively impaired the proliferation of *L. interrogans* cultures. Adding glutamine to non-physiological EMJH medium also rapidly induced a short-term proliferative boost in *L. interrogans* and increased biofilm formation. RNA-sequencing after glutamine exposure revealed transcriptional trends for increases in biosynthesis to support these phenotypes. Although ammonium has long been thought to be the sole nitrogen source for *Leptospira* spp.*,* our results demonstrate that glutamine provides a second source of nitrogen for biosynthesis in multiple strains and may act as a metabolite signal to alter *L. interrogans* physiology in ways that could influence infection. This work highlights that studying *L. interrogans* under physiological conditions is key to understanding mechanisms supporting infection and points to nitrogen assimilation as a potential target for therapies.

## Author summary

Leptospirosis is a potentially fatal disease transmitted through water and soil contaminated with pathogenic *Leptospira* bacteria. Much research is currently focused on the idea that an improved understanding of how *Leptospira* infects hosts and causes disease may inspire the development of improved therapeutics, which are urgently needed. Focusing on *Leptospira interrogans*, a clinically important species, we determined that conventional growth media are inadequate for understanding how the bacterium behaves when inside hosts. Instead, we designed an optimized formulation to mimic human blood, and we applied an underutilized technique for measuring the biochemical reactions that enable pathogen survival. These two innovations revealed that *L. interrogans* uses glutamine, an abundant nutrient in host blood and tissues, as a source of nitrogen for the production of biomolecules that are required for replication and infection. Our discovery is notable because nitrogen demands were previously thought to be met with ammonium. Treating *L. interrogans* with inhibitors of both glutamine and ammonium metabolism blocked bacterial replication. We also discovered that *L. interrogans* increases its growth rate, upregulates its expression of biosynthetic pathways, and increases its formation of biofilm when exposed to glutamine. Our results reveal the importance of glutamine in supporting the lifecycle of leptospirosis-causing bacteria.

## Introduction

Leptospirosis is a bacterial disease associated with exposure to flooding and poor sanitation [1,2]. Incidence worldwide reached an estimated annual count of one million in 2015 and continues to steadily increase across many regions, with reported fatality rates between 5–20% [3–5]. Animal studies investigating the impact of post-colonization interventions and meta-analyses of clinical studies have called into question the effectiveness of standard-of-care antibiotics, including penicillin, outside of prophylaxis [6,7]. Some of these antibiotics target specific processes in cellular metabolism (e.g., peptidoglycan biosynthesis); however, there are many other pathways also critical for replication, motility, host immune evasion, and toxin production [8–11] that have not been evaluated for therapeutic viability in leptospirosis. Metabolic interventions outside of cell wall biosynthesis have been successful in the treatment of other infectious diseases, such as tuberculosis [12]. We posit that a better understanding of the metabolic mechanisms supporting infection and pathogenicity of disease-causing *Leptospira* spp. could reveal new, urgently needed treatment strategies.

Early work demonstrated that pathogenic *Leptospira* spp., such as *Leptospira interrogans*, require exogenous fatty acids as a carbon source for biomass and energy production [13]. Similar studies revealed that carbon from fatty acids and select amino acids is used by pathogenic *Leptospira* spp. to biosynthesize proteins as well as pyrimidine and purine nucleotides [14]. In contrast, the comparative preferences for different nitrogen sources in *Leptospira* spp. are unknown. The specific nitrogen assimilation reactions and the molecules into which nitrogen is incorporated also remain unevaluated. Regardless of these uncertainties, past studies concluded that ammonium was the preferred source of nitrogen based on growth rate [14,15]. Given that protein-based toxins, nucleic acids, and polyamines all contain nitrogen and contribute to pathology and proliferation, nitrogen metabolism is likely a key driver of virulence and a potential vulnerability.

Beyond incomplete knowledge of nitrogen assimilation, our understanding of *Leptospira* metabolism is further limited by two additional factors. First, many existing conclusions have been inferred from genomics [16], transcriptomics [17,18], or proteomics [19,20] data, with the implicit assumption of correlated gene expression and metabolic activity. Metabolomics offers a better approach to assess metabolic pathways by directly measuring substrates and products. Despite this potential, it has yet to be implemented in *Leptospira* research. The second factor limiting the impact of metabolism research in *Leptospira* spp. is the widespread application of non-physiological media that do not replicate the nutrient, temperature, or osmolarity conditions of human hosts. The pitfall of this practice is exemplified by multiple reports of metabolic rewiring in response to host adaptation from a non-physiological medium [17,18,21–24]. Taken together, it is unclear whether previously reported observations related to metabolism translate accurately to *in vivo* settings.

Here, we developed approaches to overcome both of the above barriers that have hindered progress to understand and therapeutically exploit *Leptospira* spp. metabolism. Inspired by efforts in cancer research to create physiologically relevant media [25,26] and building off of recent work in *Leptospira* [27], we designed a medium that closely reflects the nutrient conditions experienced by *Leptospira* spp. in human hosts. In parallel, we also established liquid chromatography/mass spectrometry (LC/MS)-based metabolomics and isotope-tracing workflows for *Leptospira* spp. The application of our updated medium formulation and global profiles of metabolic pathways led us to the surprising observation that glutamine (Gln) is a major source of nitrogen for multiple *Leptospira* strains and that it can control the rate of proliferation and biofilm formation.

## Results

### Establishing a workflow to study *L. interrogans* metabolism

Recent work in both mammalian cells and microbes has shown that the physiology of cells cultured in conventional medium formulations with minimal components at supraphysiological concentrations does not reflect the physiology of the same cells *in vivo* [18,25,27]. Concerns over this observation led to the development of improved medium formulations containing a greater variety of small molecules at concentrations similar to average values in human plasma, such as human plasma-like medium (HPLM, Gibco, A4899101) [28,29]. Although HPLM is not intended for long-term growth without medium changes, because nutrients may be rapidly depleted by highly active cell types, it improves confidence in the clinical relevance of research findings. Notably, lipophilic metabolites normally present in human plasma are not included in the formulation of HPLM that is currently available commercially.

We aimed to develop a physiologically relevant medium for *Leptospira* culture. Because *Leptospira* spp. are thought to require fatty acids for carbon assimilation [30], which are generally accessible in human hosts, we first attempted continuous culture in HPLM supplemented with 10% fetal bovine serum (FBS) and a bovine serum albumin (BSA):oleate conjugate as lipid sources (Fig 1A). We inoculated the supplemented HPLM (sHPLM) with the commonly used strain Fiocruz L1-130 of *Leptospira interrogans* serovar (sv.) Copenhageni, which causes hepatic, pulmonary, and renal pathology in hamsters [31] and C3H/HeJ mice [32,33]. Conventionally, *Leptospira* strains are cultured at 28–31 ºC in EMJH (Ellinghausen-McCullough-Johnson-Harris) medium, a serum-devoid minimal medium. Ammonium, supplied at 4.67 mM [34], is the sole nitrogen source in EMJH, which is in 100-fold excess compared to most locations in host organisms from which *L. interrogans* can be isolated. Tween 80, a xenobiotic surfactant carrying a fatty acid mix of primarily oleate, is the carbon source. In such non-physiological conditions, the growth rate is typically 18 hours per doubling [7]. In our hands, EMJH cultures exhibited an average of 15.5 hours per doubling during exponential phase (S1A Fig). By contrast, in sHPLM cultures, we observed robust growth, reaching a maximum rate of approximately 4.5 hours per doubling when cultured at 37 ºC and 5% CO_2_. For sHPLM cultures at 30 ºC and without CO_2_ regulation, we measured a doubling time of about 6.1 hours (S1B Fig). These growth rates are about twofold greater than values obtained *in vivo* using qPCR and luminescent *L. interrogans* in guinea pig and mouse models [7,35], where host immune mechanisms would be opposing maximal replication. In our sHPLM experiments, we suspected that depletion of lipids was limiting to the maximum culture density as is expected for primary nutrients in physiological media. Indeed, by adding additional BSA:oleate conjugate to stationary phase culture, a higher maximum density was reached (Fig 1A). However, for further experiments we supplemented BSA:oleate at 2% to approximate physiological concentrations more closely without significantly diluting the amount of HPLM.

**Fig 1:**
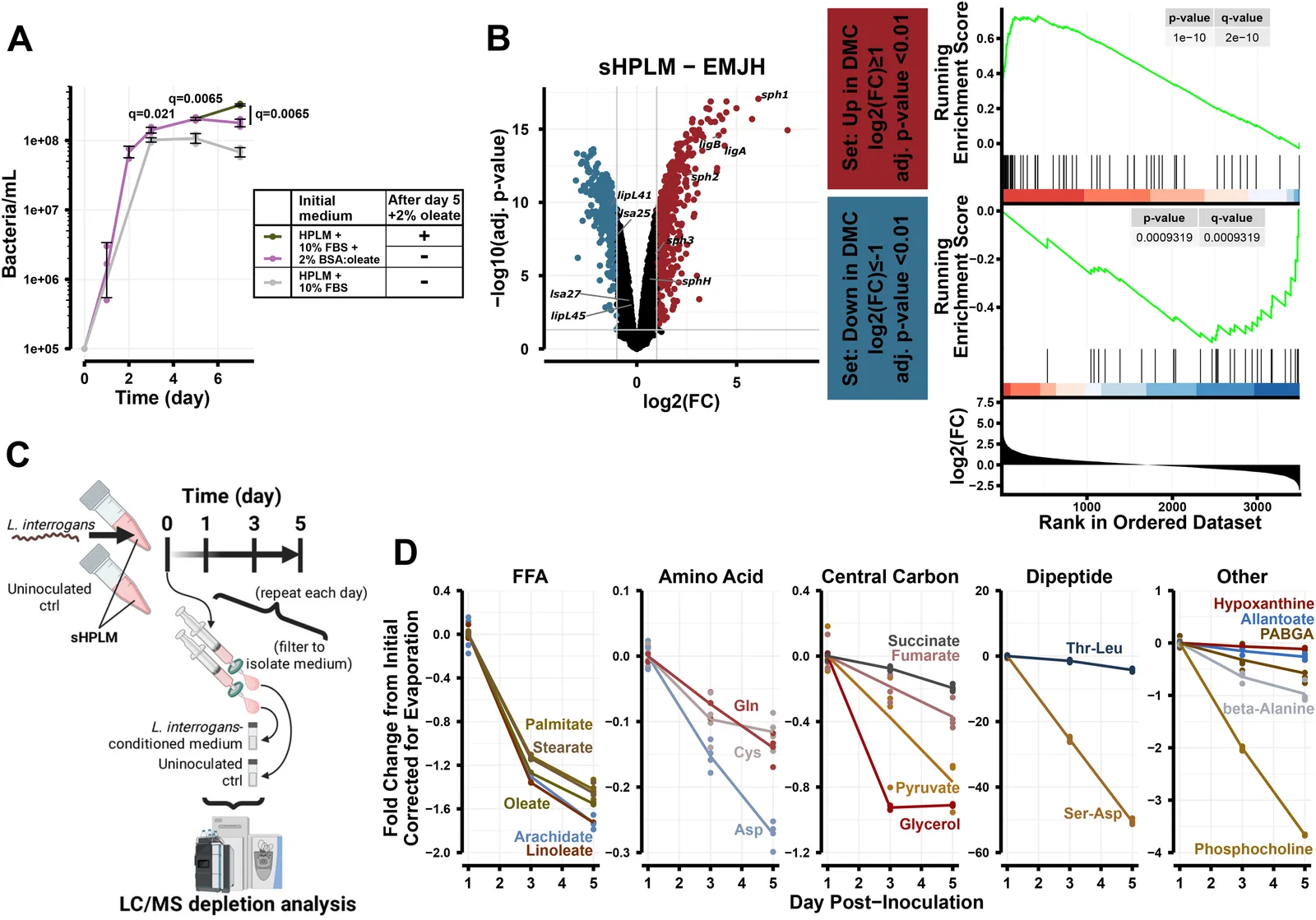
An LC/MS-based nutrient screen for *L. interrogans* using plasma-like medium. **(A)** Darkfield-counted bacterial density of *L. interrogans* sv. Copenhageni strain Fiocruz L1-130 over seven days in the indicated sHPLM mixture. Tubes (5 mL) were inoculated in triplicate at 1e5 bacteria/mL. After the day-five count, three replicates in the + 2% BSA:oleate group were spiked with an additional 2% BSA:oleate. Maximum densities: (green) total 4% BSA:oleate, 3.25e8 bacteria/mL at day seven; (purple) total 2% BSA:oleate, 2.12e8 bacteria/mL at day five; (grey) no BSA:oleate, 1.08e8 bacteria/mL at day five. **(B)** RNA-sequencing differential expression analysis. (left) DEGs from exponential phase (5e7 bacteria/mL) EMJH cultures after four hours in EMJH or sHPLM to assess early gene expression changes upon exposure to a plasma-like environment. Red: increased transcript abundance; blue: decreased. Select virulence-related genes labeled. (right) Gene set enrichment analysis of fold change-ranked transcripts from the sHPLM-EMJH comparison using gene sets formed from false-discovery rate < 0.01 and -1 ≥ log2(FC) ≥1 DEGs in the Caimano et al. dataset [18]. The green line represents running enrichment score. **(C)** Schematic for applying sHPLM and LC/MS to identify metabolites depleted over time from host-like medium in *L. interrogans* monocultures. Created in Biorender. Ward, M. H. (2026) https://BioRender.com/bp1is1s. **(D)** Metabolites with a fold change < -11% from days one to five. Sample fold change corrected against changes in uninoculated medium over time. n = 3 independent samples per group for A except for day-five +oleate, which was n = 6, and n = 4 for B and D. Asp, aspartate; BSA, bovine serum albumin; Cys, cysteine; DEGs, differentially expressed genes; DMC, dialysis membrane chamber; EMJH, Ellinghausen-McCullough-Johnson-Harris medium; FBS, fetal bovine serum; FC, fold change; FFA, free fatty acid; Gln, glutamine; Leu, leucine; LC/MS, liquid chromatography/mass spectrometry; PABGA, para-aminobenzoylglutamate; sHPLM, supplemented Human Plasma-Like Medium; Thr, threonine.

To examine the process of host adaptation and the extent to which our system aligned with other host models, we performed RNA-sequencing on *L. interrogans* four hours after switching to sHPLM (Fig 1B and S1 Table). Changes in the gene expression profile, compared to EMJH cultures, showed similarity by gene set enrichment analysis (GSEA) to a published transcriptomics study evaluating *L. interrogans* implanted in rats (Caimano et al., 2014), using the published differentially expressed genes as a gene set [18]. We observed specific changes in gene expression in sHPLM-cultured *L. interrogans* Fiocruz L1-130 consistent with host adaptation and virulence, including in *ligA*, *ligB*, *sph1/2/3*, *sphH*, *lsa27*, *lsa25*, *lipL41*, and *lipL45* (S1C Fig). The virulence-related gene differential expression observed in sHPLM cultures was similar to the Caimano et al. dataset as well as an additional transcriptomics study of *L. interrogans* evaluated in hamster liver tissue three days after infection (Giraud-Gatineau et al., 2026) [24] (S1D Fig).

Widening the scope of analysis by using the “biological process” ontology gene sets, GSEA and overrepresentation analysis in four-hour sHPLM cultures revealed a trend for downregulation of biosynthetic metabolism, energy generation, and translation, as well as a trend for upregulation of protein import, fatty acid biosynthesis, and transcription (S1E Fig). While metabolic genes specifically identified by Giraud-Gatineau et al. had differing patterns between the three transcriptomics datasets (S1F Fig), this may align with the timing of measurements and differences in the nutrient environment of bacteria between the studies (i.e., plasma compared to liver and peritoneal cavity). Consequently, we confirmed that virulence-related transcriptional trends (represented by increases in *sph1*, *ligA*, *ligB*, and *bfr*) held past the four-hour mark in sHPLM-cultured *L. interrogans* Fiocruz L1-130, by measuring expression 48 hours after the EMJH-sHPLM switch (S1G Fig).

With a strategy in place for culture under host-like conditions, we next sought to explore the metabolic properties of *L. interrogans* by profiling which nutrients were utilized. We inoculated *L. interrogans* Fiocruz L1-130 into sHPLM at mid-exponential phase and extracted metabolites from filtered conditioned medium after one, three, and five days of culture for LC/MS-based metabolomics (Fig 1C). We identified 111 metabolites in fresh and culture-conditioned sHPLM and measured the relative concentration of each as a function of time (S2 Table). We note that few metabolites showed evidence of significant change at early timepoints, indicating that the metabolic effect of low-density cultures on their surroundings is relatively small. Those metabolites that did decrease over time relative to uninoculated replicates were assumed to be nutrients taken up by bacteria. We report 19 putative nutrients that were depleted by *L. interrogans* Fiocruz L1-130 when cultured under host-like conditions (adjusted p-value < 0.05 and fold change decrease of greater than 11%, Fig 1D). The fold change cutoff was defined by a split in the distribution of fold change values from day one to day five (S2A Fig). Consistent with prior reports using EMJH [36], we observed the depletion of several fatty acids and glycerol. Notably, we also identified the depletion of three amino acids: cysteine (Cys), aspartate (Asp), and glutamine (Gln). The observation that not every amino acid was significantly depleted (S2B Fig) suggests roles for Cys, Asp, and Gln outside of proteinogenesis, which, if required for survival under host-like conditions, could potentially be exploited to design novel therapeutics.

### Glutamine and ammonium are major nitrogen sources for *L. interrogans*

Nitrogen utilization by *L. interrogans* has been studied far less than carbon metabolism although it is equally important to virulence mechanisms, such as protein production for host evasion. We were interested in the possibility that one or more of our putative nutrients might provide nitrogen to *L. interrogans*. To evaluate the contribution of candidate nitrogen sources to the nitrogen pool of *L. interrogans* relative to previously established sources such as ammonium and urea [37], we turned to stable isotope tracing.

We spiked sHPLM with a given ^15^N-labeled nutrient (the tracer) at 50% of the existing non-labeled nutrient concentration in HPLM, for a starting value of 33% labeling in the extracellular metabolite pool. This amount was expected to minimize the perturbation of metabolic steady state while still providing enough isotope label to trace. We collected intracellular and extracellular samples with a combined workflow for analysis of tracer assimilation (Fig 2A), which relied upon syringe and vacuum filtration (further details in Methods). Our approach separated intracellular and extracellular metabolites faster than conventional methods that rely on centrifugation, which is critical for accurate analysis of rapid metabolic processes. As expected, when vacuum-filtration was applied to isolate intracellular metabolites, inoculated cultures showed much higher amino acid abundances than uninoculated controls without bacteria (Fig 2B and 2C). Thus, this method enabled separation of the intracellular and extracellular amino acids, even those with high medium concentrations that would be expected to contaminate the intracellular sample otherwise.

**Fig 2:**
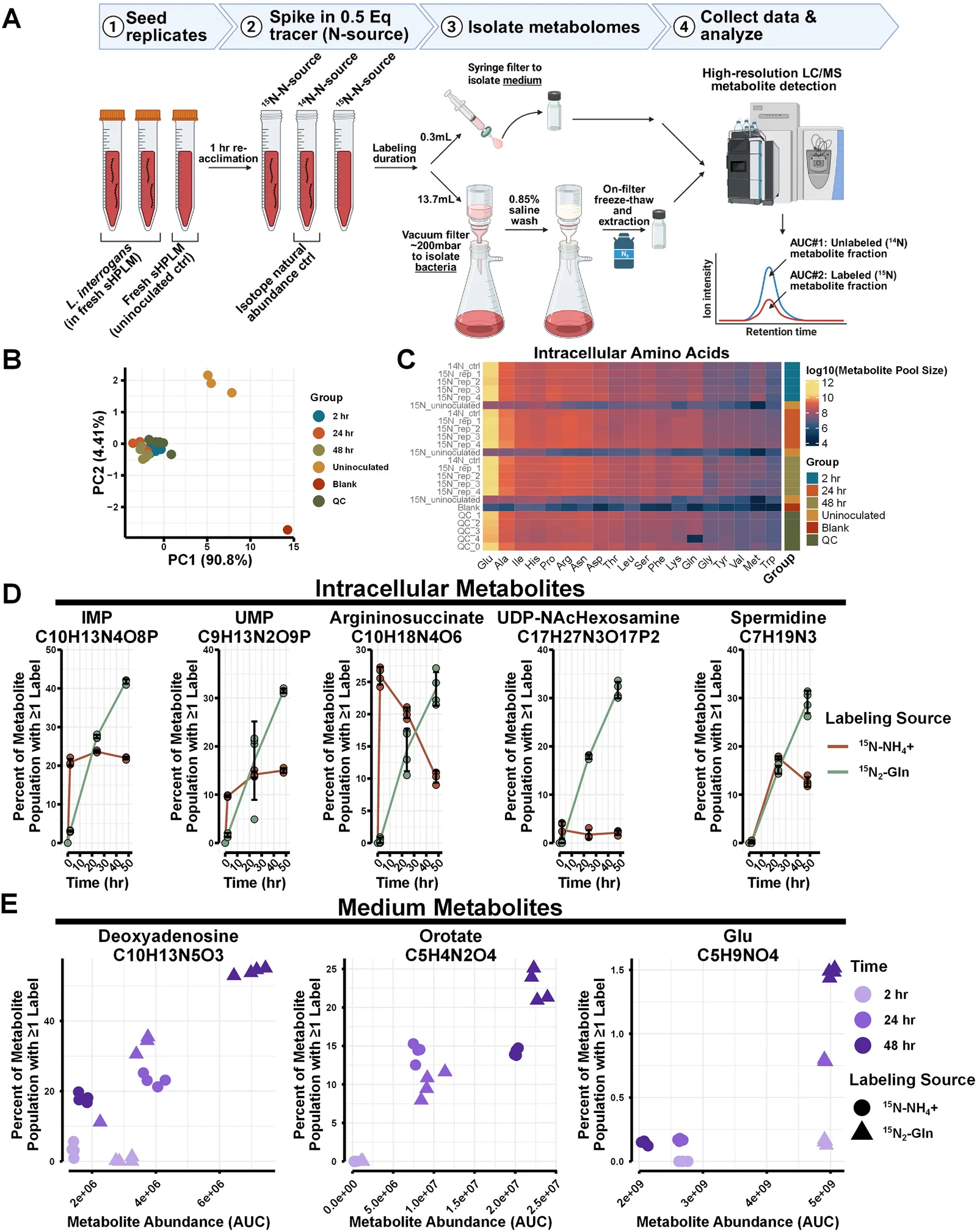
Ammonium and glutamine are *L. interrogans’* major nitrogen sources in physiological conditions. **(A)** Schematic for combined stable isotope-tracing analysis of intracellular and medium metabolites. Created in BioRender. Ward, M. H. (2026) https://BioRender.com/b0eifft. **(B)** Principal component analysis plot and **(C)** heatmap of summed pool sizes of 19 detected amino acids in individual replicate samples processed through the vacuum filtration workflow. Intracellular *L. interrogans* metabolomes were sampled at 2, 24, and 48 hours. Uninoculated samples from the same timepoints (each shown below the respective five inoculated replicates) are filtered culture medium without bacteria. Blank is extraction solvent only. QC is a pooled sample used as a reference material throughout data collection. **(D)** Percentage of given metabolite populations in *L. interrogans* Fiocruz L1-130 that have at least one labeled nitrogen from host-like medium. ^15^N is derived from ammonium or Gln after 2, 24, or 48 hours of labeling in sHPLM spiked to a concentration of 33.3% labeled nitrogen source. Metabolites are representatives of the pathways: purine, pyrimidine, arginine, glycosylation, and polyamine biosynthesis. The timepoint at zero hours was imputed at 0% labeling. **(E)** Medium metabolite percentage labeling and relative abundance from the same experiments in panel D. n = 4 independent samples per group for B-E. Ala, alanine; Arg, arginine; Asn, asparagine; Asp, aspartate; AUC, area under the curve; Cys, cysteine; Gln, glutamine; Glu, glutamate; Gly, glycine; His, histidine; Ile, isoleucine; IMP, inosine monophosphate; LC/MS, liquid chromatography/mass spectrometry; Leu, leucine; Lys, lysine; Met, methionine; Phe, phenylalanine; Pro, proline; Ser, serine; sHPLM, supplemented Human Plasma-Like Medium; Thr, threonine; Trp, tryptophan; Tyr, tyrosine; Val, valine; UDP-NAcHexosamine, uridine diphosphate N-acetylhexosamine; UMP, uridine monophosphate.

After incubation with a ^15^N-labeled tracer, evidence of bacterial uptake and assimilation was assessed by the time-dependent accumulation of other biomolecules bearing ^15^N label. These molecules were built through biochemical incorporation of one or more nitrogen atoms from the tracer (S3A Fig). In time-resolved isotope tracing, the labeling percentage tends to increase until reaching a plateau. The parameters of this curve for a given biomolecule indicate the rate of tracer incorporation, the size of the metabolite pool, and the contribution of the tracer to biosynthesis relative to other sources of nitrogen.

As a benchmark for comparison, we first cultured bacteria in sHPLM spiked with ^15^N-ammonium. Probable glutamine synthetase (GlnA) activity, the expected reaction assimilating ammonium, led to the labeling of predominantly one of two nitrogen atoms in glutamine (S3B Fig), and we also detected time-dependent increases of ^15^N labeling in downstream metabolites across several biosynthetic pathways (Fig 2D and S4). Our data provided evidence that host-derived ammonium fuels nucleotide biosynthesis (both purines and pyrimidines), amino acid biosynthesis, glycosylation, and polyamine biosynthesis in host-like conditions. Analysis of medium metabolites similarly showed increased labeling of nucleic acids and amino acids over time, with some also increasing in total abundance as though secreted (Fig 2E). Consistent with the idea that these labeled metabolites in the medium were secreted during the experiment rather than leaked from bacterial lysis, other metabolites (e.g., hexose phosphates and NAD^+^) were only detected in intracellular samples (S3C Fig). These data suggest that select newly synthesized nucleic acids and amino acids are secreted by *L. interrogans* Fiocruz L1-130, potentially in the form of a short peptide or extracellular DNA.

We next compared these results against an identical experiment using ^15^N_2_-Gln as the tracer, chosen because its reported concentration in the HPLM formulation was sufficiently high to support biomass production in high-density cultures. Remarkably, we observed that in any given biomolecule population, the percentage that was built with Gln-derived nitrogen after one day of labeling was comparable or even higher than from ^15^N-ammonium in most observed areas of nitrogen metabolism (Fig 2D and S4). There was no pathway that exclusively utilized either nitrogen source, though some metabolites showed differential labeling dynamics. This lack of exclusivity in nitrogen sourcing could indicate metabolic redundancy. We also note that although the synthesis of intracellular Gln using nitrogen from exogenous ammonium generated primarily singly labeled Gln (M + 1), little of this isotopologue was synthesized with nitrogen from exogenous ^15^N_2_-Gln (S3B Fig). This supports the possibility that Gln is directly imported and incorporated into *L. interrogans* metabolism rather than first needing to be deaminated/deamidated to release ammonium. Similar to the ammonium labeling experiment, select metabolites in the medium were also labeled in a time-dependent manner with ^15^N_2_-Gln-derived nitrogen (Fig 2E).

We performed similar isotope-tracing experiments with ^15^N-Asp, due to its depletion from sHPLM, and ^15^N_2_-urea, a previously proposed nitrogen source [37]. In both cases, only minimal percentages of intracellular metabolites from sHPLM cultures were labeled (S3D Fig). These data suggest that Asp and urea are only minor sources of nitrogen under physiological conditions. Additionally, to assess the conservation of Gln assimilation in a different strain of *L. interrogans*, we measured labeling from exogenous ^15^N_2_-Gln at the 24-hour timepoint in *L. interrogans* sv. Manilae strain L495 and observed significant labeling of intracellular metabolites (S3E Fig).

We conclude that, contrary to conventional wisdom drawn from studies in non-physiological media, *L. interrogans* does not rely solely upon ammonium for nitrogen assimilation. Instead, we show that Gln is an important source of nitrogen when grown in host-like conditions with access to a variety of potential nitrogen sources. We also present a metabolic map of the major biosynthetic pathways active in *L. interrogans* Fiocruz L1-130 under host-like conditions with data from which qualitative pathway activity estimates can be drawn for future vulnerability analysis and the development of therapeutic interventions (S4 Fig).

### Glutamine fuels robust replication of multiple *Leptospira* spp. and can be targeted as a metabolic hub

Our tracing studies yielded a model of parallel Gln and ammonium utilization for biosynthesis under host-like conditions, indicating that metabolic redundancy in nitrogen assimilation could be possible. Therefore, we next sought to discern whether either ammonium or Gln were required for proliferation and represented a nutrient vulnerability. For this purpose, we replicated the HPLM formulation in-house and evaluated the proliferation of *L. interrogans* sv. Copenhageni strain Fiocruz L1-130 when only ammonium or Gln were provided as a primary nitrogen source, as well as without urea, aspartate, and calcium nitrate. To largely eliminate FBS-derived nitrogen sources, dialyzed FBS was used at 5% instead of regular FBS at 10%. In this formulation, minor nitrogen sources (e.g., Asp, urea) provided a small, 0.8-fold benefit to *L. interrogans* Fiocruz L1-130 proliferation above the negative control, whereas adding 550 µM Gln or ammonium chloride elicited robust proliferation (>threefold above negative control, ~ seven hours/doubling) (Fig 3A). Adding Gln and ammonium chloride together failed to elicit more rapid proliferation than ammonium chloride alone. These results were largely replicated in *L. interrogans* sv. Manilae strain L495, except that the minor nitrogen sources did not contribute significantly to proliferation and there was a small (0.14-fold) additive benefit to having both Gln and ammonium chloride (S5A Fig). The slightly reduced replicative rate fueled by Gln alone may be explained by kinetic differences in assimilation revealed by our isotope tracing (Fig 2). These results demonstrate that Gln and ammonium are largely redundant and sufficient for robust proliferation. Further, we conclude that other metabolites in the HPLM formulation, including those shown to be depleted such as Cys, were not sufficient nitrogen sources.

**Fig 3:**
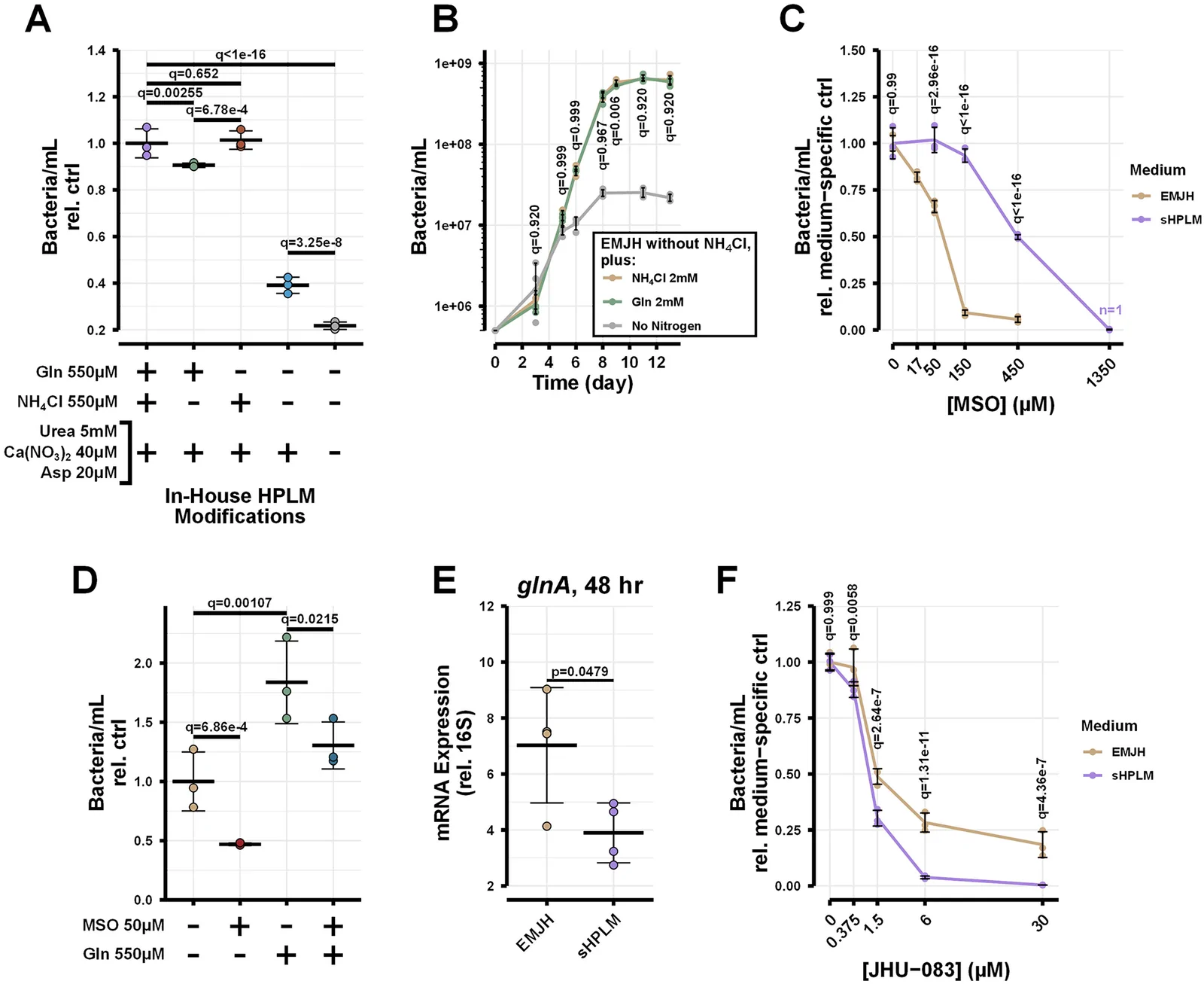
Nitrogen assimilation redundancy and metabolic adaptation in sHPLM cultures. **(A)** Proliferation assay measured by darkfield microscopy at two days from initial density of 5e5 bacteria/mL with *L. interrogans* Fiocruz L1-130. Base medium is in-house synthesized HPLM at published concentrations, lacking ammonium chloride, Gln, Asp, calcium nitrate, and urea. To all groups, 5% dialyzed FBS and 2% BSA:oleate were added. Density normalized to fully supplemented condition. **(B)** Growth curve from 5e5 bacteria/mL in EMJH lacking ammonium chloride, supplemented with either 2 mM ammonium chloride, Gln, or vehicle. q-values shown are comparisons between the + Gln and + ammonium chloride groups. **(C)** Proliferation assay measured at two days in the indicated culture medium, inoculated at 2.5e6 bacteria/mL with the indicated MSO concentrations. At 1350 µM MSO in sHPLM, only one replicate had detectable bacteria. X-axis in square-root scale. **(D)** Proliferation at two-day endpoint in standard EMJH (4.67 mM ammonium) supplemented with 50 µM MSO and/or 550 µM Gln. Inoculated at 5e6 bacteria/mL. **(E)** Cultures grown in EMJH medium were switched to either fresh EMJH or sHPLM at 8e6 bacteria/mL and pelleted 48 hours later for RNA isolation. Expression of glutamine synthetase, *glnA*, is shown. At endpoint, cultures were confirmed at densities of approximately 5e7 bacteria/mL. Expression relative to the housekeeping gene 16S rRNA of *L. interrogans*. **(F)** Dose-response proliferation assay inoculated at 2.5e6 bacteria/mL, measured at two-day endpoint. Response to varying JHU-083 dosages. X-axis in square-root scale. All panels show results in *L. interrogans* sv. Copenhageni strain Fiocruz L1-130. n = 3 independent samples per group for A, C-D and F; n = 4 for B and E. Welch’s t-test performed for E. A-D, and F multiple contrasts, see Statistics in Methods. Asp, aspartate; Ca(NO_3_)_2_, calcium nitrate; EMJH, Ellinghausen-McCullough-Johnson-Harris medium; Gln, glutamine; MSO, methionine sulfoximine; NH_4_Cl, ammonium chloride; sHPLM, supplemented Human Plasma-Like Medium.

We then evaluated Gln assimilation in bacteria grown at 30 ºC in specially formulated EMJH with either Gln or ammonium chloride as the sole nitrogen source. Under these conditions, ammonium and Gln were each sufficient to fuel proliferation at relatively equivalent rates in *L. interrogans* sv. Copenhageni strain Fiocruz L1-130 (Fig 3B). *L. weilii* sv. Topaz strain LT2116, a pathogenic species, and *L. biflexa* sv. Patoc strain Patoc-1, a nonpathogenic species, were also able to replicate using only Gln in EMJH (S5B-S5C Fig), suggesting that Gln catabolism is conserved across multiple *Leptospira* species.

With information on metabolic remodeling in *L. interrogans* grown under host conditions from our RNA-sequencing and isotope tracing, we subsequently set out to reveal vulnerable points in the mechanism of nitrogen assimilation by inhibiting key steps. Specifically, we tested the ammonium assimilation inhibitor methionine sulfoximine (MSO) as well as the Gln utilization inhibitor JHU-083 (S5D Fig).

The *L. interrogans* genome lacks an annotated homolog of glutamate dehydrogenase. Assuming glutamate dehydrogenase activity is absent, the primary mechanism to assimilate ammonium nitrogen into biomass is through the synthesis of Gln from ammonium and glutamate by GlnA (S5D Fig). To test this model we used MSO, an irreversible inhibitor of glutamine synthetase (GlnA). To validate its mechanism of action, we evaluated proliferation with and without inhibitor in *L. interrogans* Fiocruz L1-130 that had been passaged in EMJH medium lacking the ammonium chloride component and either complemented with 2 mM ammonium chloride or supplemented with 2 mM Gln. MSO blunted proliferation in EMJH cultures of *L. interrogans* Fiocruz L1-130 when grown on ammonium but not when grown on Gln (S5E Fig), suggesting that MSO acts on-target and that GlnA is indeed critical to EMJH cultures for the metabolic usage of ammonium. However, proliferation in sHPLM cultures was approximately nine-fold less sensitive to inhibition by MSO than standard EMJH cultures (Fig 3C). The most likely metabolite confounder to the effect of MSO, glutamate [38], did not replicate this resistance when added to EMJH cultures (S5F Fig). Further, supplementation with Gln and MSO together also did not block inhibition by MSO (Fig 3D), although Gln did independently increase endpoint density.

A more compelling explanation for MSO resistance in sHPLM is that metabolic remodeling unique to sHPLM-cultured *L. interrogans* leads to upregulation of alternate pathways for ammonium assimilation (i.e., challenging the assumption of reliance on GlnA activity). To explore this line of reasoning, we examined the transcriptional expression of glutamine synthetase in sHPLM cultures 48 hours after switching from EMJH medium. We observed evidence of *glnA* downregulation between EMJH and sHPLM cultures (Fig 3E). Querying our RNA-seq dataset from short-term sHPLM exposure revealed similar *glnA* downregulation, along with expression changes in associated nitrogen metabolism proteins and regulators (S5G Fig). These data suggest changes in ammonium assimilation during host adaptation. Considering these results together with the resistance to MSO in sHPLM, and rapid appearance of M + 1 glutamate from ^15^N-ammonium (S4 Fig), we surmise that changes in ammonium assimilation may result in a reliance on GlnA-independent mechanisms to fuel proliferation.

We also tested the susceptibility of *L. interrogans* Fiocruz L1-130 to the 6-diazo-5-oxo-L-norleucine (DON) prodrug JHU-083, which inhibits Gln-utilizing enzymes in mammalian cells [39]. We treated cultures with a range of JHU-083 concentrations and observed that proliferation was blunted by over half in the low micromolar range, with significantly higher sensitivity in sHPLM cultures (Fig 3F). These data further underscore the metabolic differences between sHPLM and EMJH cultures. *L. weilii* sv. Topaz strain LT2116, a pathogenic strain, showed even higher JHU-083 sensitivity in EMJH (S5H Fig). We conclude that JHU-083 has promising antibacterial activity against two pathogenic *Leptospira* strains.

### Glutamine exposure induces a rapid proliferative bloom in *L. interrogans*

We found that supplementing EMJH with Gln at physiological concentrations yielded higher density cultures of *L. interrogans* Fiocruz L1-130, implying that Gln increased their proliferation rate (Fig 3D). Before exploring the mechanism underlying this effect and its relation to the increased proliferation rate of sHPLM cultures, we examined whether it was conserved in two other *Leptospira* strains. We found that the addition of 550 µM Gln to EMJH cultures of *L. interrogans* sv. Manilae strain L495 did boost its proliferation at one day post-exposure. In contrast, the effect was not observed for the non-pathogenic, saprophytic *L. biflexa* sv. Patoc strain Patoc-1 (Fig 4A). This provided evidence that, unlike Gln assimilation, the proliferative response to Gln is not conserved across both pathogenic and nonpathogenic *Leptospira* spp.

**Fig 4:**
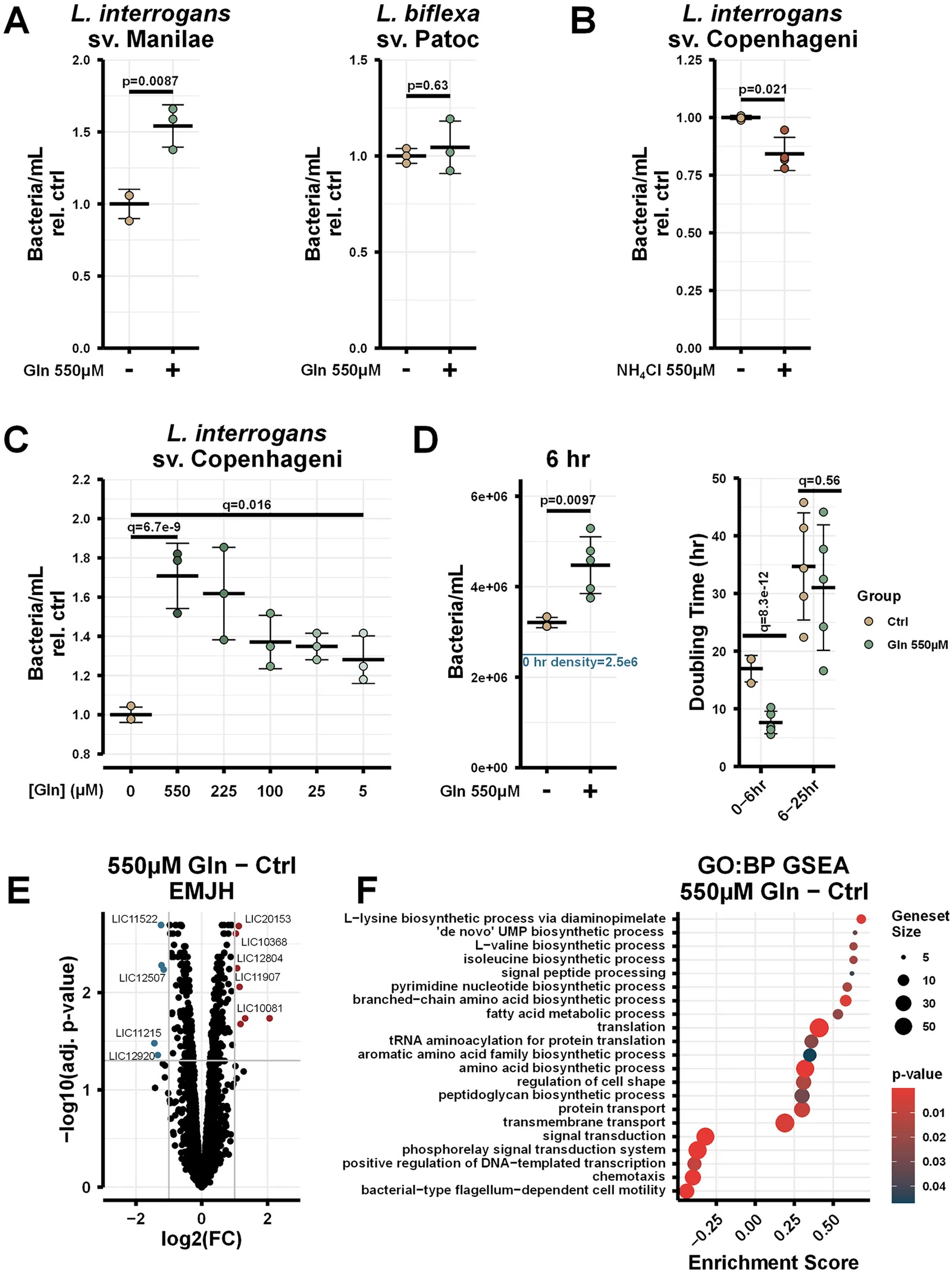
Exogenous glutamine stimulates a short-term proliferative boost in pathogenic *L. interrogans.* **(A)** EMJH cultures of *L. interrogans* sv. Manilae strain L495 (pathogenic) and *L. biflexa* sv. Patoc strain Patoc-1 (nonpathogenic) supplemented with 550 µM Gln after seeding at 1e6 bacteria/mL, with proliferation assessed after one day by darkfield microscopy. **(B)** Relative density after two days of proliferation from 2.5e6 bacteria/mL in EMJH cultures of *L. interrogans* sv. Copenhageni strain Fiocruz L1-130 supplemented with an additional 550 µM ammonium chloride. **(C)** Relative density of *L. interrogans* Fiocruz L1-130 after two days of proliferation from 2.5e6 bacteria/mL of EMJH cultures given the indicated amounts of Gln. **(D)** (left) Relative density measurement six hours after seeding *L. interrogans* Fiocruz L1-130 at 2.5e6 bacteria/mL in EMJH supplemented with either vehicle or 550 µM Gln, demonstrating the early timescale of the effect. (right) From the same culture, doubling time between the 0-6 hour interval and the 6-25 hour interval. **(E)** RNA-sequencing differential expression. RNA was isolated from *L. interrogans* Fiocruz L1-130 exponential phase (5e7 bacteria/mL) EMJH cultures after four hours in EMJH+550 µM Gln versus EMJH (Gln-Ctrl). Data from the EMJH group is shared with the plot in Fig 1B. Red indicates increased transcript abundance, blue indicates decreased. **(F)** Gene set enrichment analysis of expression trends in the Gln-Ctrl comparison. n = 3 independent samples for A-C; n = 5 for D; n = 4 for Ctrl and n = 3 for Gln in E-F. Welch’s t-test performed for A-B and D (left); for C and D (right) multiple contrasts, see Statistics in Methods. EMJH, Ellinghausen-McCullough-Johnson-Harris medium; FC, fold change; Gln, glutamine; GO:BP, gene ontology: biological process; GSEA, gene set enrichment analysis; NH_4_Cl, ammonium chloride; sHPLM, supplemented Human Plasma-Like Medium.

We next determined that the effect of Gln on proliferation was medium-dependent, as providing additional Gln atop the 550 µM already present in sHPLM did not accelerate the proliferation rate of *L. interrogans* Fiocruz L1-130 (S6A Fig). Given that we identified a metabolic role for Gln in sHPLM cultures, we then tested the possibility that the effect of Gln on proliferation in EMJH cultures was due to extra nitrogen supply. However, supplementing EMJH cultures with 550 µM ammonium did not phenocopy Gln (Fig 4B). This result suggests that the proliferative effect we observed occurs independent of the utilization of Gln-derived nitrogen to build biomass.

We suspected that Gln might be acting as a metabolite signal inducing accelerated proliferation separate from its role as an important nutrient. While carbon and nitrogen sources are generally required at high concentrations to fuel rapid proliferation, signaling molecules can elicit effects at low concentrations. Consistent with the latter, the addition of Gln at concentrations as low as 5 µM stimulated a disproportionately high increase in proliferation (~1.25-fold above control) (Fig 4C). The Gln effect also appeared to be rapid and short-lived. A decrease in doubling time manifested within six hours of Gln exposure, and thereafter the difference was muted (Fig 4D and S6B). Metabolomics analysis revealed that the amount of Gln depleted within one day was minimal (S6C Fig), and thus consumption of the available Gln could not explain the transience of the effect. The low eliciting dosage, timescale, transience, and lack of correspondence to Gln levels in the medium support a model of Gln acting as a signaling metabolite.

To better understand its role as a potential signaling molecule, we assessed other aspects of *L. interrogans* Fiocruz L1-130 physiology that might be impacted by Gln. We examined biofilm formation and observed evidence that 550 µM Gln increased crystal violet-stained biofilm at seven days and 14 days. In contrast, equimolar ammonium chloride failed to promote biofilm formation (S6D Fig), similar to the trend we observed in proliferation assays. We also performed RNA-sequencing on EMJH cultures four hours after exposure to 550 µM Gln. Strikingly, we observed few genes meeting our fold change thresholds comparing Gln-exposed *L. interrogans* Fiocruz L1-130 to vehicle controls (Fig 4E and S3 Table). We instead turned to GSEA and observed the upregulation of genes associated with various biosynthetic metabolic pathways, including some leading to cell wall synthesis, and translation. These results aligned with the phenotype of increased proliferation. There was also a general downregulation of signaling, motility, and chemotaxis-related genes (Fig 4F). Using genes with a gene ontology annotation or annotated in NCBI with a protein product related to transcriptional regulation, we identified possible regulatory proteins mediating the effect of Gln, according to the logic that many transcription regulation mechanisms are also self-regulating (S6E Fig). Overall, these results illustrate that environmental Gln can inform components of *L. interrogans* physiology that are involved in the pathogen’s lifecycle.

## Discussion

In this study, we developed two resources to advance metabolism research in *Leptospira* spp., both of which may be translated to the study of other microorganisms. First, we established a growth medium referred to as sHPLM, which approximates the human environment inhabited by infectious agents with a bloodstream phase. This medium induced phenotypes in *L. interrogans* sv. Copenhageni strain Fiocruz L1-130 similar to those observed *in vivo*. Second, we designed a workflow to perform metabolomics and stable isotope tracing on *L. interrogans* cultures and spent medium that enabled us to identify active metabolic pathways and qualitatively estimate the rates of nutrient transformation into biomass. The application of these resources revealed that Gln is a key source of nitrogen for two *L. interrogans* strains under host-like conditions, where it is used to a similar extent as ammonium. Further investigation revealed that nitrogen assimilation can be chemically inhibited to block bacterial proliferation, and that Gln also has effects similar to an environmental signal, inducing proliferation, biofilm formation, and biosynthetic gene expression.

The disparity in phenotypes of *L. interrogans* cultured in EMJH and sHPLM emphasizes the need to shift culture techniques in microbiology to be more physiologically relevant and account for the strong influence of the environment on metabolic activity [40,41]. Indeed, there is an increasing call to use physiological media for the study of infectious disease [41–44]. This trend has seen success in research on mammalian cells, where using HPLM has revealed key aspects of cancer therapy [25] and immune cell activation [45]. The physiological accuracy of measurements made with leptospires cultured in this formulation was critical to our conclusions about host-relevant metabolism. We highlight that sensing of the environmental cues in sHPLM was sufficient to transcriptionally induce many virulence processes and metabolic alterations independent of physical interaction with host cells. As such, sHPLM may help deconvolute complex host-adaptation mechanisms with greater ease.

The complex formulation of sHPLM, which offers many potential nutrients, permits a more representative assessment of the nutrient preferences of *L. interrogans* in hosts than can be achieved in minimal media such as EMJH, where only the bacterium’s capacity to adapt under duress can be evaluated [37,46]. This distinction is key to understanding what metabolites *L. interrogans* actually obtains from its host during infection, which may influence pathogenesis. From our list of putative nutrients, we focused on Gln due to its high concentration and its central role in nitrogen metabolism and host physiology [47]. Beyond Gln, our screen revealed several other metabolic characteristics of *L. interrogans* Fiocruz L1-130 that were not pursued here. In line with conventional thinking, our results suggest that this strain relies upon fatty acids. Thus, future work should examine which specific lipids are preferred under physiological conditions and the possible mechanistic connections of lipid consumption to the appearance of lipid-filled foamy macrophages in leptospirosis [48], or to injury-induced kidney steatosis [49,50]. Our screen also demonstrated Asp and pyruvate depletion by *L. interrogans* Fiocruz L1-130. Asp-derived nitrogen contributed minimally to biosynthesis, which is evidence against its utilization as a nitrogen or carbon source [46] and leaves its true role unknown. Prior work in several bacterial species demonstrated that pyruvate neutralizes reactive oxygen species and other toxic oxidative products [51], including natural byproducts of bacterial metabolism [52]. As *L. interrogans* relies on oxidative metabolism, future studies could assess whether this mechanism supports bacterial replication in the host. These connections to existing literature reinforce that our metabolomics-based nutrient screen can yield additional insights as a resource to the research community.

The metabolomics experiments performed herein represent, to our knowledge, the most thorough molecular interrogation of *L. interrogans* metabolism to date. Unlike many prior studies that have made inferences about *Leptospira* metabolism from indirect measurements, we assess metabolism within bacterial cells directly by measuring the substrates and products of enzymatic reactions via LC/MS. Our method paves the way for more comprehensive multi-omics study designs in *Leptospira* cultures to better understand pathogenesis. Two other studies have used LC/MS in the context of leptospirosis to profile infected animal urine and plasma, but these primarily yielded information about the host response to infection [53,54]. It is also important to note that our approach for sample isolation with vacuum filtration builds off of work in other liquid-cultured [55,56] and solid-cultured species [57]. We previously employed a similar protocol to study gut microbe-host metabolic interactions [58], but here the washing step was adjusted to effectively remove most metabolites from the complex formulation of sHPLM. This alteration is of critical importance when performing metabolomics on bacteria cultured in any nutrient-rich medium.

With this metabolomics workflow, we revisited the longstanding conclusion from *L. interrogans* Pomona that Gln could not serve as a sufficient nitrogen source unless first autoclaved to release ammonium [15]. Here, using several species and serovars of varying pathogenicity, we demonstrated successful culture in medium lacking ammonium but containing unautoclaved Gln. By using ^15^N-tracers, we confirmed that Gln-derived nitrogen is used to produce biomass for proliferation and metabolite secretion. Metabolites secreted by *L. interrogans* Fiocruz L1-130 could be contributions to biofilms, which are known to contain a substantial amount of extracellular DNA [59], or they could be indicative of phage release. Our results necessitate reconsideration of the published conclusion [60,61] that ammonium is the only effective nitrogen source. Host innate immune cells, including macrophages, also rely upon Gln [62], so our findings could imply metabolic competition in infected tissues. Our use of JHU-083 to target Gln metabolism, similar to past uses in *Mycobacterium tuberculosis* infection to limit bacterial burden and enhance macrophage responses [63], showed promising inhibition of *L. interrogans* Fiocruz L1-130 and *L. weilii* LT2116 proliferation. This provides motivation for future preclinical studies using JHU-083 or the similar DON prodrug Sirpiglenastat that is already in phase 2 clinical trials (NCT07249372).

Based on our data, Gln may also have a non-nutrient role in *L. interrogans*. Its rapid effect on proliferation and the transcriptome is consistent with a Gln-sensing mechanism. The abundance of Gln relative to ammonium varies between stages of the known lifecycle of pathogenic *Leptospira* spp. For example, Gln is high and ammonium is low in mammalian blood due to ammonia toxicity [64], whereas the reverse is true in urine and possibly in the urine-contaminated soil and water that transmits *L. interrogans* [65]. It is noteworthy that the temporary proliferative bloom was observed in two pathogenic strains but not a nonpathogenic *Leptospira* strain. Should Gln responsiveness correlate with the ability to colonize animal hosts, we speculate that this could indicate a mechanism that evolved alongside the emergence of host-infecting *Leptospira* spp. to engage context-appropriate physiological responses and promote infection, similar to the virulence-activating effect of Gln on *Listeria monocytogenes* [66]. Further, because *L. biflexa* is saprophytic and not known to colonize hosts, there is likely no advantage to respond to Gln. That said, the mechanism and factors influencing the response to Gln remain to be discovered, and further work should deconvolute the role of Gln from other metabolites in the induction of host adaptation.

We acknowledge several limitations of our study. First, we tested Gln assimilation in four strains of *Leptospira*. While the phenotype was conserved among the strains examined, there is wide diversity within the genus that may affect the generalizability of this metabolic trait, as well as the observed effects of Gln on proliferation and biofilm formation. Similarly, as exemplified by the *L. weilii* strain used herein, the ability to grow in sHPLM is not universal. It may be that some pathogenic strains such as *L. weilii* LT2116 do not thrive in blood, which could explain prior work showing a low blood burden following intravenous infection of C3H/HeJ mice [67]. Second, our transcriptomics comparison to the Caimano et al. and Giraud-Gatineau et al. datasets must be experimentally contextualized. It is possible that our comparisons were affected by the different timescales and conditions under which the bacteria were assessed, especially for metabolic genes that tend to be highly responsive to the bacterial environment. Further, we performed transcriptomics after four hours of sHPLM exposure to reveal the early hallmarks of bacterial adaptation to the host environment. The results may not reflect the transcriptome of *L. interrogans* Fiocruz L1-130 in many other experiments presented here, in which bacteria had been acclimated to sHPLM for several days to ensure metabolic steady state. A third limitation of our work is the comparison of phenotypes in EMJH and sHPLM cultures, which have several different variables (e.g., temperature and CO_2_) that could influence results in addition to the nutrient composition. We note that one such phenotype, JHU-083 sensitivity, can so far only be viewed as an antibacterial effect until activity studies with this drug in *L. interrogans* have confirmed its inhibition of Gln-utilizing reactions. Finally, we acknowledge that the full effect of Gln, and its mechanism and strain-dependence, remain incompletely characterized. An investigation beyond the proliferation assays used herein, including biofilm assays and a comparison of transcriptional markers, may provide additional insight into how this metabolite affects physiology in pathogenic and nonpathogenic strains.

Notwithstanding, using an innovative physiological medium and isotope-tracing workflow to derive critical metabolic information, our data point to Gln as a key metabolite fueling *L. interrogans* replication and influencing pathogen physiology. We hope that the present work will help inspire the development of new therapeutic strategies for severe leptospirosis targeted at vulnerabilities in nitrogen metabolism.

## Methods

### Materials and handling of bacterial strains

*L. interrogans* sv. Copenhageni strain Fiocruz L1-130, *L. biflexa* sv. Patoc strain Patoc-1, *L. interrogans* sv. Manilae strain L495, and *L. weilii* sv. Topaz strain LT2116 were generously provided by Dr. Jenifer Coburn, Medical College of Wisconsin, and Dr. Mathieu Picardeau, Institut Pasteur. Pathogenic strains were at three passages from an animal host upon transfer and were used to no more than three additional passages to maintain virulence. Pathogenic strains were thawed rapidly in a 37 ºC water bath in Hornsby-Alt-Nally (HAN) medium, prepared as previously described [68], containing 100 µg/mL 5-fluorouracil (MilliporeSigma, F6627). Nonpathogenic *Leptospira* were thawed in liquid EMJH medium, which was composed of 90% Base (BD Difco, 279410) suspended in Milli-Q water and autoclaved, then mixed with 10% Enrichment (BD Difco, 279510). Strains were frozen slowly in aliquots of 0.25 mL culture medium in a CoolCell chamber (Corning, 432001). Pathogenic strains were cultured either in liquid EMJH at 30 ºC without shaking in conical tubes ~75% filled with medium or at 37 ºC and 5% CO_2_ in sHPLM or HAN in the same format, as required for successful culture in the respective media, and containing 100 µg/mL 5-fluorouracil. sHPLM culture medium was prepared by adding 10% 0.22 µm-filtered FBS (ThermoFisher, 16000044 or Corning 35–015-CV) and 2% BSA:oleate conjugate (Cayman Chemical, 29557) to HPLM (Life Technologies, A4899101). When custom-synthesized, sHPLM included dialyzed FBS at 5% (Cytiva, SH3007902). All bacterial strains were handled in a biosafety cabinet with BSL-2 procedures, respiratory protection, and restricted access. The metabolic inhibitors tested included methionine sulfoximine (MilliporeSigma, M5379), and JHU-083 (Cayman Chemical, 40642). Compounds supplemented to culture medium included ammonium chloride (MilliporeSigma, 213330), ^15^N-ammonium chloride (Cambridge Isotope Laboratories, NLM-467), L-glutamine (MilliporeSigma, 49419), ^15^N_2_-L-glutamine (Cambridge Isotope Laboratories, NLM-1328), urea (MilliporeSigma, U5128), ^15^N_2_-urea (Cambridge Isotope Laboratories, NLM-233), L-aspartic acid (MilliporeSigma, A8949), ^15^N-L-aspartic acid (Cambridge Isotope Laboratories, NLM-718), calcium nitrate (MilliporeSigma, C1396), and L-glutamic acid (MilliporeSigma, G8415).

### Isotope tracing

*L. interrogans* cultures, passaged for at least two days in sHPLM, were counted and centrifuged at 3700 RCF and 30 ºC for 30 min, and the pellets were resuspended in 0.25 mL sHPLM per 40 mL of culture pelleted. During this time, fresh sHPLM was prepared and warmed to 37 ºC, and 14 mL was removed for uninoculated replicates for each timepoint. The bacterial suspension was then recounted and inoculated into sHPLM at 1e8 bacteria/mL for two-hour isolation, at 2.5e7 bacteria/mL for 24-hour isolation, and at 1e7/mL for 48-hour isolation and split into 15 mL conical tubes. The suspensions were rested for one hour in the incubator to allow metabolism to equilibrate, before each tube was sequentially spiked 1:1000 at ten-minute intervals with either ^15^N-labeled tracer or its ^14^N-unlabeled equivalent (uninoculated replicates were spiked with labeled tracer) and gently mixed by serological pipette twice. After the labeling time elapsed, a selection of inoculated tubes were counted to ensure a density around 1e8 bacteria/mL, and intracellular and medium samples were taken from each tube sequentially.

### Metabolite extraction from medium

After gentle mixing, 0.35 mL of culture was removed with a syringe, then filtered through a 0.1 µm filter (Millipore, SLVVR33RS), which sterilized media down to the limit of detection by darkfield microscopy and a Petroff-Hausser chamber. Filtered medium samples were then diluted 1/10 in LC/MS-grade 2:2:1 acetonitrile:methanol:water pre-chilled at -25 ºC, vortexed one min, incubated at -25 ºC for one hour to precipitate proteins, snap-frozen in liquid nitrogen, and stored at -80 ºC. Before analysis, samples were thawed on ice, centrifuged at 18000 RCF and 4 ºC for ten min, and the supernatant transferred to glass LC vials.

### Metabolite extraction from cells

A vacuum filtration setup was prepared with a 500 mL Erlenmeyer filtration flask connected to a vacuum pump (KNF, UN811KTP) pulling -200 mbar. Capping the flask was the PELL magnetic filter funnel (Cytiva, 4247) with a pre-activated (autoclaved milli-Q water) 47 mm 0.1 µm PTFE filter (Millipore, JVWP04700). *L. interrogans* cultures were poured over filters under vacuum, and afterwards 10 mL 0.85% saline (pre-warmed to 37 ºC) dispensed around the sides of the reservoir to ensure movement of bacteria to the filter area. The filter was then removed and transferred to the bottom half of the Restek Diskcover-47 (VWR, R24020), which replaced the PELL filter holder unit on the vacuum trap. This next unit has a larger effective filtration area, upon which the filter was washed with 7 mL saline and gently blotted dry on the underside with a VWR Light-Duty tissue wipe. The filter was then transferred to a 50 mL conical tube containing 1.2 mL LC/MS-grade 2:2:1 acetonitrile:methanol:water, which had been pre-chilled on dry ice, then carefully vortexed for 30 sec without folding the filter, snap-frozen in liquid nitrogen for ten min (during which time the next sample was isolated), and transferred to dry ice until all samples were isolated. To process, conical tubes were sequentially thawed and bath-sonicated for three min at 20 ºC (without heating), vortexed and shaken for 30 sec, snap-frozen in liquid nitrogen for three min, and re-sonicated for three min at 20 ºC, after which the liquid was removed to 1.5 mL tubes. These tubes were then vortexed briefly, snap frozen in liquid nitrogen for three min, then sonicated for three min and incubated at -25 ºC overnight. It was confirmed that this procedure killed all bacteria. The following day, samples were centrifuged at 18000 RCF and 4 ºC for 15 min and approximately 92.5% of the liquid transferred to fresh 1.5 mL tubes for drying on a SpeedVac concentrator. Dried pellets were resuspended in 60 µL 2:1 acetonitrile:water, subjected to two cycles of five min bath-sonication (no heating) and one min vortexing in order to ensure maximum resuspension, then incubated at 4 ºC overnight. Finally, samples were centrifuged at 18000 RCF and 4 ºC for ten min and the supernatant transferred to glass LC vials. This extraction protocol was previously optimized for broad coverage of the polar metabolome [58,69]. However, no single protocol is optimized to isolate the entire metabolome [70], and this method may not capture volatile metabolites and lipids.

### Liquid chromatography/mass spectrometry

For data collection, metabolite extracts were analyzed on either a Thermo Q-Exactive Orbitrap or an ID-X Orbitrap Mass Spectrometer interfaced with a Thermo Vanquish Horizon LC system using an iHILIC-(P) Classic 2.1 mm  ×  100 mm, 5 μm column (HILICON, 160.102.0520) with an iHILIC-(P) Classic Guard column (HILICON, 160.122.0520) attached. The column temperature was set to 45 °C. Mobile phase A was 95% water, 5% acetonitrile, 20 mM ammonium bicarbonate, 0.1% ammonium hydroxide solution (25% in water), and 5 µM medronic acid. Mobile phase B was 95% acetonitrile and 5% water. Each sample was injected (6 µL of intracellular and 2 µL of medium metabolite extract) and eluted with a linear gradient: at 250 µL/min 0–1 min 90% B, 1–12 min 35% B, 12-12.5 min 25% B, 12.5–14.5 min 25% B, 14.5–15 min 90% B, 15–20min at 400 µL/min and 90% B, and 20–22 min at 250 µL/min and 90% B for re-equilibration. A small volume (typically 7–10 µL) was taken from each sample and mixed together in a single glass vial to create a reference material. Replicate injections of the reference material were done at regular intervals throughout the data collection sequence for quality control (labeled as “QC”) [71]. MS/MS fragmentation data were collected using targeted methods at 20 eV. Metabolites were identified based on matching accurate mass, retention time, and MS/MS data to authentic reference standards from our in-house library or from curated databases for level one or two identification confidence, respectively [72]. Chromatograms for selected metabolites were manually integrated in Skyline Daily (version 22.2.1.425).

### Isotope-tracing analysis

Peak areas from manual integration in Skyline Daily (version 22.2.1.425) were loaded into R (version 4.4.2). Natural isotope abundance correction was performed in R using the github release of the accucore package [73] (version 0.3.1), with a modified nitrogen_isotope_correction function to eliminate unnecessary nontracer corrections when complete resolution of the labeled nitrogen isotopologue by high-resolution mass spectrometry was obtained (confirmed in Skyline Daily). Isotopologue peak areas were normalized as a percent of the summed intensity of all isotopologue peaks per sample and metabolite, and plotted as: (i) a summed total percentage of all labeled (M > 0) isotopologues, (ii) as the percentage of each isotopologue, or (iii) as a percentage of total ^15^N nitrogen atoms in the metabolite pool with correction for the initial tracer labeling percentage to reflect the true percent of nitrogen atoms derived from the tracer.

### Depletion analysis from metabolomics data

Peak areas from manual integration in Skyline Daily (version 22.2.1.425) were loaded into R (version 4.4.2). First, metabolites were normalized as zero-centered fold change relative to the average signal from day-one samples. Uninoculated control measurements were moderated to reduce measurement error by using linear models created from metabolite-specific peak area data and obtaining predicted outputs for days one, three, and five, which were then normalized as zero-centered fold change relative to the predicted value from the day-one sample. Fold changes in inoculated samples were then corrected for changes in the uninoculated controls by subtracting day-matched uninoculated values from inoculated sample values. Fold changes from day one to day five were plotted as a histogram to choose a suitable cutoff, and metabolites meeting this cutoff with a q-value<0.05 as determined by Welch’s t-test with Benjamini-Hochberg adjustment were plotted in R. Metabolomics data have been deposited in the NIH Metabolomics Workbench repository under study ID ST005044.

### Density measurements and proliferation assay

Bacterial density was assessed with a Petroff-Hausser chamber (Electron Microscopy Sciences, 63512–24) loaded with 2.5 µL culture, with dilution in HAN, EMJH, or deionized water as needed, visualized in darkfield on a Leica DM500 microscope (NCI Inc., 13613215). Between four and eight grid sections of the 1 mm center square were counted at 400X total magnification, averaged, and multiplied by 25 grid squares by 50000 mL^-1^ to obtain the bacterial culture concentration (bacteria/mL). For proliferation assays, bacteria were resuspended to the indicated density in fresh medium and aliquoted at 3–4 mL each to 5 mL tubes (Midsci, C2535). Tube caps were parafilm-wrapped and incubated for the indicated times, whereupon culture was gently mixed to resuspend sedimented bacteria and counted.

### Biofilm assay and microscopy

*L. interrogans* culture was dispensed at 150 µL/well in 96-well plates (Midsci, 781962) with outer wells filled with deionized water and incubated for seven days or 14 days. Medium was carefully removed, and 175 µL 10% formalin added and incubated 30 min at room temperature before removal. Each well was then washed twice with 200 µL PBS (Gibco, 10-010-049), and 175 µL crystal violet solution (0.1%, MilliporeSigma, V5265) was added and incubated for ten min. Each well was then washed twice with 200 µL PBS, and the plate was dried overnight. Individual wells were imaged using a Leica THUNDER imaging system at 5x and 10x with automatic stitching together. Images were imported into ImageJ (version 1.50i), clipped to the boundaries of the well, color thresholding applied (HSB: Hue 39–255, Saturation 50–255, Brightness 99–199), and area measured relative to 1 mm scale bars.

### RNA isolation

*L. interrogans* cultures were centrifuged at 3700 RCF and 30 ºC for 30 min, then the pellet was resuspended in 1 mL Trizol (Invitrogen,15596018) and snap-frozen in liquid nitrogen for storage at -80 ºC. For processing, samples were thawed on ice, centrifuged for five min at 18000 RCF and 4 ºC, mixed with 0.2 mL chloroform by inverting several times, and then incubated at room temperature for three min. Samples were centrifuged for 15 min at 12000 RCF and 4 ºC and the clear aqueous phase transferred to a new tube, to which was added 0.5 mL cold isopropanol. Tubes were inverted gently to mix and incubated for ten min on ice before being centrifuged for 15 min at 18000 RCF and 4 ºC. The supernatant was removed and the RNA pellet resuspended in 75% ethanol, vortexed briefly, and centrifuged again for ten min at 18000 RCF and 4 ºC. The ethanol was then carefully removed, the pellet dried for five min at room temperature and resuspended in nuclease-free water by pipetting and heating at 55 ºC for five min. RNA was then treated with DNase as per the instructions of the DNA-free kit (ThermoFisher, AM1906) and quantified with a NanoDrop instrument (ThermoFisher).

### RNA-sequencing and analysis

RNA-sequencing was performed by the Genome and Technology Access Center (GTAC) at Washington University in St. Louis. RNA integrity was quantified with the Agilent Bioanalyzer or 4200 Tapestation. cDNA libraries were prepared with an input of 500 ng to 1 µg of total RNA, as follows. RNA was fragmented according to the FastSelect protocol (Qiagen) in reverse transcriptase buffer (94 ºC for five min, 75 ºC for two min, 70 ºC for two min, 65 ºC for two min, 60 ºC for two min, 55 ºC for two min, 37 ºC for five min, and 25 ºC for five min), with ribosomal RNA depleted during the process. cDNA was synthesized from remaining mRNA with the SuperScript III RT enzyme (Life Technologies) and random hexamers according to the manufacturer’s instructions. ds-cDNA was formed with a second strand reaction. The product library was blunt ended and had an A base added to the 3’ ends before then ligating Illumina sequencing adapters. The library was amplified for 15 cycles with primers incorporating unique dual index tags. Sequencing was performed on an Illumina NovaSeq X Plus using paired end reads extending 150 bases, with Illumina’s bcl2fastq software used for base calling and demultiplexing setting a threshold of a maximum of one mismatch in the indexing read. Reads were aligned with STAR (version 2.7.11b) to the *Leptospira interrogans* sv. Copenhageni str. Fiocruz L1-130 GCF_000007685.1 (ASM768v1) primary assembly. Gene counts were calculated from uniquely aligned and unambiguous fragments with Subread:featureCount (version 2.0.8). Sequencing performance was evaluated based on total versus uniquely aligned reads, features detected, the ribosomal fraction, known junction saturation, and read distribution over known gene models using RSeQC (version 5.04).

Differential gene count was evaluated with the R/Bioconductor (version 4.4.0) package EdgeR and Limma using TMM normalization size factors to adjust for library size. Reads for ribosomal genes and those with a lower count than one count per million in at least three samples were excluded. A Limma generalized linear model was fitted using weighted likelihoods calculated from gene and sample-wise mean-variance relationships and the voomWithQualityWeights function. This model was used for differential expression analysis, with Benjamini-Hochberg false-discovery rate adjustment applied for p-values. One sample in the EMJH+Gln group was determined to be a statistical outlier and was removed from the analysis, leaving three independent replicates. The data are stored under NCBI BioProject number PRJNA1401465 and GEO accession GSE316384.

Gene set analyses were performed in R (version 4.4.2) using the packages UniProt.ws (version 2.46.1), ClusterProfiler (version 4.14.6), and functions from the package HypeR (version 2.0.0) modified to employ a two-tailed version of the ks.test function. Gene sets were downloaded from the QuickGO website [74] with filters: taxon = 173 (include descendants), gene product = proteins. GO terms were further filtered to exclude sets with fewer than five genes. The remaining library of sets contained many sets with few detected genes, which skewed post-hoc p-value correction. Thus, p-values are reported for gene set analysis.

### RT-qPCR

RNA was first reverse transcribed to cDNA with the iSCRIPT cDNA Synthesis Kit (Bio-Rad, 1708891) according to the manufacturer’s protocol and a Bio-Rad S1000 Thermal Cycler (Bio-Rad, 1852148). cDNA was diluted by 1/4 in nuclease-free water. Primers were synthesized by IDT. The following sequences were used: 16S-F 5’-AGGTAAAGATTCGATAGGAGCG-3’, 16S-R 5’-GTAGAACCGCTTTCTGGGG-3’, glnA-F 5’-CTCTTCTTGCCTTGTCTCCGT-3’, glnA-R 5’-GTGGGGTTCTTTCTCACGCT-3’, ligA-F 5’- TCCTGAGCGAAGGTCTTACACTACAA-3’, ligA-R 5’- TCTCCAAATGCAAGAGCCGTTAC-3’, sph1-F 5’- GGAAAGATTCGATCAGCCAGAGA-3’, sph1-R 5’- GCGATAAATAAGATCCGCACTCC-3’, ligB-F 5’- AGGTAACTCCGGCACAATTGATTT-3’, ligB-R 5’- GTCACGTCTTGTACGGAATGATCC-3’, bfr-F 5’- TGAAGATCGGGCACTCCGT-3’, bfr-R 5’- CGATAGACGAAATGAAACACGCC-3’. Primers were resuspended to 100 nM in nuclease-free water and diluted to working concentrations of 10 nM. qPCR reactions were assembled in 96-well reaction plates (Applied Biosystems, 4346906) as follows: 2 µL diluted cDNA, 0.5 µL each forward and reverse primer, 5 µL Power SYBR PCR Master Mix (Applied Biosystems, 4367659), and 2 µL nuclease-free water. Each plate was centrifuged briefly at 2000 RCF, then run on an Applied Biosystems StepOne Plus instrument (software version 2.3) in technical duplicate. Gene expression was calculated as ΔCt relative to the 16S RNA measurement for each sample.

### Statistical analysis

Statistical analyses were performed in R (version 4.4.2). Data were analyzed by two-tailed Welch’s T-test when only one pairwise contrast was made. When multiple pairwise contrasts were made, groupwise data were assessed for heteroscedasticity with a Bartlett test, cutoff p < 0.1, and either a hetero- or homoscedastic generalized least squares model created using the gls function from the nlme package (version 3.1-166). Prespecified pairwise contrasts from the model were tested using the glht function from the multcomp package (version 1.4-26), analogous to specified contrasts tested on an ANOVA model [75]. Post-hoc correction using the Benjamini-Hochberg method was then applied to produce q-values. Effect sizes were calculated with the effectsize package (version 1.0.0). A pairwise difference was considered statistically significant when p < 0.05 or q < 0.05 and hedge’s “g” effect size was ≥ 2.5. Data in all point plots are presented as mean + /- 1 standard deviation. All plots were created in R, using packages: ggplot2 (version 3.5.1), NatParksPallettes (version 0.2.0), ggh4x (version 0.2.8), plyr (version 1.8.9), dplyr (version 1.1.4), ComplexHeatmap (version 2.22.0), circlize (version 0.4.17), ggrepel (version 0.9.6), enrichplot (version 1.26.6), and reshape2 (version 1.4.4). Schematics were created using either Inkscape or Biorender.

## Supporting information

S1 Fig sHPLM cultures mimic the *in vivo* transcriptome. (A) Growth curve measured by darkfield microscopy of *L. interrogans* sv. Copenhageni strain Fiocruz L1-130 grown at 30 ºC in liquid EMJH from 1e5 bacteria/mL. (B) Growth curve in sHPLM at either 30 ºC without CO_2_, or 37 ºC and 5% CO_2_ from a density of 1e5 bacteria/mL. (C) CPM transcript expression in selected virulence-related genes from EMJH cultures switched to EMJH or sHPLM for four hours. (D) Heatmap of log2(FC) comparison values for select virulence-related genes as defined in Giraud-Gatineau et al.[24] calculated between EMJH cultures and a host-adapted condition. Host-adapted conditions include sHPLM measured in this study, dialysis membrane chambers in rats after ten days of implantation (Caimano et al., 2014) [18], and liver tissue of hamsters three days post-infection (Giraud-Gatineau et al., 2026) [24]. (E) (top) GSEA of expression trends in the sHPLM-EMJH comparison. (middle and lower) Gene set ORA in q < 0.05 and log2(FC)>1 or log2(FC)<-1 genes in the sHPLM-EMJH comparison. (F) Heatmap of log2(FC) comparison values for select metabolism-related genes described in Giraud-Gatineau et al.[24] calculated between EMJH cultures and a host-adapted condition, as defined above for panel D. (G) mRNA expression measured by RT-qPCR of *sph1* (sphingomyelinase), *ligA*, *ligB*, and *bfr* (bacterioferritin) relative to the 16S rRNA in *L. interrogans* Fiocruz L1-130. Cultures grown in EMJH were switched to either fresh EMJH or sHPLM at 8e6 bacteria/mL and pelleted 48 hours later for RNA isolation. At endpoint, cultures were confirmed at densities of approximately 5e7 bacteria/mL. n = 4 independent samples per group for A, C, E, G; n = 3 for B. For details related to the multiple contrasts in B, see Statistics in Methods. Statistical difference in C determined with EdgeR and Benjamini-Hochberg correction. Welch’s t-test performed for G. CPM, counts-per-million; DT, doubling time; EMJH, Ellinghausen-McCullough-Johnson-Harris medium; FC, fold change; GO:BP, gene ontology: biological process; GSEA, gene set enrichment analysis; ORA, overrepresentation analysis; sHPLM, supplemented human plasma-like medium.
(PDF)

S2 Fig sHPLM cultures consume select metabolites. (A) (upper) Histogram of metabolite total fold changes from 111 detected metabolites from days one through five. (lower) Zoomed-in plot of histogram showing -11% threshold. (B) Line plots of amino acid fold changes over time. n = 4 independent samples for A-B. Ala, alanine; Arg, arginine; Asn, asparagine; Asp, aspartate; Cys, cysteine; Gln, glutamine; Glu, glutamate; Gly, glycine; His, histidine; Ile, isoleucine; Leu, leucine; Lys, lysine; Met, methionine; Phe, phenylalanine; Pro, proline; Ser, serine; Thr, threonine; Trp, tryptophan; Tyr, tyrosine; Val, valine.
(PDF)

S3 Fig Stable isotope tracing of nitrogen sources in physiologically cultured *L. interrogans.* (A) Example breakdown of relative abundance of isotopologues (mass isotopologue distribution) for IMP over time, showing gradual incorporation of ^15^N into downstream metabolism. (B) Mass isotopologue distribution of Gln for *L. interrogans* sv. Copenhageni strain Fiocruz L1-130 labeled from either exogenous ammonium or Gln. Note that some medium contamination may lead to overestimation of M + 2 label from exogenous Gln and underestimation of M + 1 and M + 2 label from ammonium. (C) Medium and intracellular metabolite extracted ion chromatograms for *m/z* corresponding to dGuanosine, NAD + , and hexose phosphate. Retention time (x-axis) in minutes. (D) Percentage of given metabolite populations that have at least one labeled nitrogen from host-like medium. The source of ^15^N was ^15^N-Asp or ^15^N_2_-urea after 2, 24, and 48 hours of labeling in sHPLM containing a concentration of 33.3% labeled nitrogen source. Metabolites are representative of initial assimilation (Glu), purine biosynthesis (adenine), and arginine biosynthesis (Arg), with the criteria that they must be detected in both experiments. Urea tracing was not performed at 48 hours. Asp labeling data are from the experiment shown in Fig 2B-2C. (E) Percentage of given metabolite populations in *L. interrogans* sv. Manilae strain L495 that had at least one labeled nitrogen from host-like medium. The source of ^15^N was ^15^N_2_-Gln after 24 hours of labeling in sHPLM containing a concentration of 33.3% labeled Gln. Metabolites are representatives of the pathways: purine, pyrimidine, arginine, glycosylation, and polyamine biosynthesis. Data in A-B are from experiments shown in Fig 2. n = 4 independent samples per group in A, B, D, E. Asp, aspartate; Arg, arginine; dGuanosine, deoxyguanosine; Gln, glutamine; Glu, glutamate; IMP, inosine monophosphate; NAD + , nicotinamide adenine dinucleotide; UDP-NAcHexosamine, uridine diphosphate N-acetylhexosamine; UMP, uridine monophosphate.
(PDF)

S4 Fig Metabolic map of nitrogen utilization in *L. interrogans.* Mapping of nitrogen labeling through detected metabolites in sHPLM-cultured *L. interrogans* sv. Copenhageni strain Fiocruz L1-130, showing active pathways and reactions. Based on isotope labeling data, where ammonium chloride or Gln served as tracers. Each red arrow indicates a single enzymatic step along a pathway. Some metabolites were not detected and were omitted. Other metabolites may have missing data when not detected at a specific timepoint. Percent labeling of nitrogen atoms calculated based on mass isotopologue distributions for each metabolite and divided by 1/3 to reflect the true percentage of nitrogen derived from each tracer. The zero-hour timepoint was imputed at 0% labeling. n = 4 independent samples per tracer per timepoint. AA, amino acid; ADP, adenosine diphosphate; ADP-ribose, adenosine diphosphate ribose; aKG, alpha-ketoglutarate; Ala, alanine; AMP, adenosine monophosphate; Arg, arginine; Asn, asparagine; Asp, aspartate; CarbamoylAsp, carbamoyl aspartate; Carbamoyl-P, carbamoyl phosphate; CDP, cytidine diphosphate; CMP, cytidine monophosphate; CTP, cytidine triphosphate; dAdenosine, deoxyadenosine; dAMP, deoxyadenosine monophosphate; dCytidine, deoxycytidine; dGuanosine, deoxyguanosine; FAD, flavin adenine dinucleotide; FGAR, 5’-phosphoribosyl-N-formylglycinamide; GDP, guanosine diphosphate; Gln, glutamine; Glu, glutamate; Glucosamine6P, glucosamine-6-phosphate; Gly, glycine; GMP, guanosine monophosphate; GTP, guanosine triphosphate; His, histidine; IMP, inosine monophosphate; Ile, isoleucine; Leu, leucine; Lys, lysine; NAD + , nicotinamide adenine dinucleotide; N-AcGlu, N-acetylglutamate; N-AcOrnithine, N-acetylornithine; Phe, phenylalanine; PRPP, phosphoribosyl pyrophosphate; Ser, serine; Thr, threonine; TMP, thymidine monophosphate; Tyr, tyrosine; UDP, uridine diphosphate; UMP, uridine monophosphate; UDP-hexose, uridine diphosphate hexose; UDP-NAcHexosamine, uridine diphosphate N-acetylhexosamine; UTP, uridine triphosphate; Val, valine.
(PDF)

S5 Fig Chemical inhibition, nutrient deprivation, and medium effects on nitrogen metabolism. (A) Proliferation assay measured by darkfield microscopy at two days from initial density of 2.5e6 bacteria/mL (*L. interrogans* sv. Manilae strain L495). Base medium is in-house synthesized HPLM at published concentrations, lacking ammonium chloride, Gln, Asp, calcium nitrate, and urea. To all groups, 5% dialyzed FBS and 2% BSA:oleate were added. Density normalized to fully supplemented condition. (B) Growth curve of *L. weilii* sv. Topaz strain LT2116 from initial density of 5e5 bacteria/mL in custom EMJH formulated without ammonium chloride and re-supplemented with either 2 mM ammonium chloride, Gln, or no nitrogen source. q-values shown are comparisons between the + Gln and + ammonium chloride groups. (C) Growth curve as in panel B, with *L. biflexa* sv. Patoc strain Patoc-1. q-values shown are comparisons between the + Gln and + ammonium chloride groups. (D) Points of chemical intervention in presumed pathway of nitrogen assimilation. (E) Proliferation assay of *L. interrogans* sv. Copenhageni strain Fiocruz L1-130 grown in custom EMJH formulated without ammonium chloride and re-supplemented with either 2 mM ammonium chloride or Gln and treated with 50 µM MSO, a glutamine synthetase inhibitor. Density measured two days after inoculation at 2.5e6 bacteria/mL. (F) Proliferation assay of *L. interrogans* Fiocruz L1-130 EMJH cultures given 50 or 450 µM MSO and 80 µM L-glutamic acid, measured at four-day endpoint from inoculation of 2.5e6 bacteria/mL. (G) CPM transcript expression of selected nitrogen assimilation-related genes from EMJH cultures switched to EMJH or sHPLM for four hours. Data are from the same experiment presented in Fig 1B. (H) Dose-response proliferation assay at two-day endpoint in response to varying JHU-083 dosages in *L. weilii* sv. Topaz strain LT2116. X-axis in square-root scale. n = 3 independent samples per group for A-C, E-F, and H; n = 4 for G. For A-C and E-F multiple contrasts, see Statistics in Methods. Statistical difference in G determined with EdgeR and Benjamini-Hochberg correction. Asp, aspartate; Ca(NO_3_)_2_, calcium nitrate; CPM, counts-per-million; aKG, alpha-ketoglutarate; EMJH, Ellinghausen-McCullough-Johnson-Harris medium; Glu, glutamate; Gln, glutamine; MSO, methionine sulfoximine; NH_4_Cl, ammonium chloride; sHPLM, supplemented Human Plasma-Like Medium.
(PDF)

S6 Fig The effect of glutamine on *L. interrogans* physiology. (A) Proliferation assay of *L. interrogans* sv. Copenhageni strain Fiocruz L1-130 sHPLM cultures given the indicated amounts of Gln (in addition to the 550 µM already present in HPLM), with density measured by darkfield microscopy at day three from an inoculated density of 1e6 bacteria/mL. (B) Calculated doubling time from the data presented in Fig 3D. Counts taken at two and five days. (C) LC/MS measurements of relative Gln concentration over time in EMJH cultures supplemented with 550 µM Gln. Normalized to average signal in uninoculated controls at the same timepoints to account for evaporation. (D) Crystal violet-stained biofilm quantitation at seven and 14 days after inoculation of 2.5e6 bacteria/mL in a 96-well plate with vehicle, 550 µM Gln, or 550 µM ammonium chloride. (E) CPM transcript expression in EMJH cultures four hours after switching to fresh EMJH, EMJH+550 µM Gln, or sHPLM in q < 0.05 genes with gene ontology annotations or protein products containing the keywords “sigma factor,” “DNA binding,” “DNA-binding,” “transcription factor,” “transcriptional regulator,” or “transcriptional repressor.” All panels show data in *L. interrogans* Fiocruz L1-130. n = 3 independent samples per group in A-B and Gln group of E, n = 4 in C and Ctrl and sHPLM groups of E, n = 10 in D. For multiple contrasts in A-D, see Statistics in Methods. Statistical difference in E determined with EdgeR and Benjamini-Hochberg correction. CPM, counts per million; EMJH, Ellinghausen-McCullough-Johnson-Harris medium; Gln, glutamine; GO:MF, gene ontology: molecular function; LC/MS, liquid chromatography/mass spectrometry; NH_4_Cl, ammonium chloride; sHPLM, supplemented Human Plasma-Like Medium; UI, uninoculated.
(PDF)

S1 Table Differentially expressed genes in *L. interrogans* Fiocruz L1-130, EMJH and sHPLM. Genes with differential mRNA expression (Benjamini-Hochberg adjusted p-value<0.05 and -1 > log2(FC)>1) from *L. interrogans* sv. Copenhageni strain Fiocruz L1-130 incubated for four hours in fresh EMJH or sHPLM. FC, fold change; sHPLM, supplemented Human Plasma-Like Medium.
(CSV)

S2 Table Detected metabolites and fold change from day one to day five. Metabolites detected in conditioned sHPLM. “diffmeans” represents total change from day-one average to day-five average. “BH_adj_p_value” indicates the Benjamini-Hochberg corrected p-value. The -11% cutoff was determined by histogram separation of fold changes (see S2A Fig). sHPLM, supplemented Human Plasma-Like Medium.
(CSV)

S3 Table Differentially expressed genes in *L. interrogans* Fiocruz L1-130, EMJH and EMJH+550 µM glutamine. Genes with differential mRNA expression (Benjamini-Hochberg adjusted p-value<0.05 and -1 > log2(FC)>1) from *L. interrogans* sv. Copenhageni strain Fiocruz L1-130 incubated for four hours in fresh EMJH or EMJH supplemented with 550 µM Gln. FC, fold change; Gln, glutamine.
(CSV)

S4 Table All non-repository data included in study. Data shown in all plots, with each plot as a separate sheet.
(XLSX)
