# Supplementary material for: Glutamine from plasma-like medium fuels nitrogen metabolism and influences proliferation in Leptospira interrogans
Source: PLoS Pathog. 2026 Oct 8;22(10):e1014534. doi: 10.1371/journal.ppat.1014534 (PMC13649119; doi:10.1371/journal.ppat.1014534)
Supplement: Supplementary file 1 [file ppat.1014534.s001.pdf]

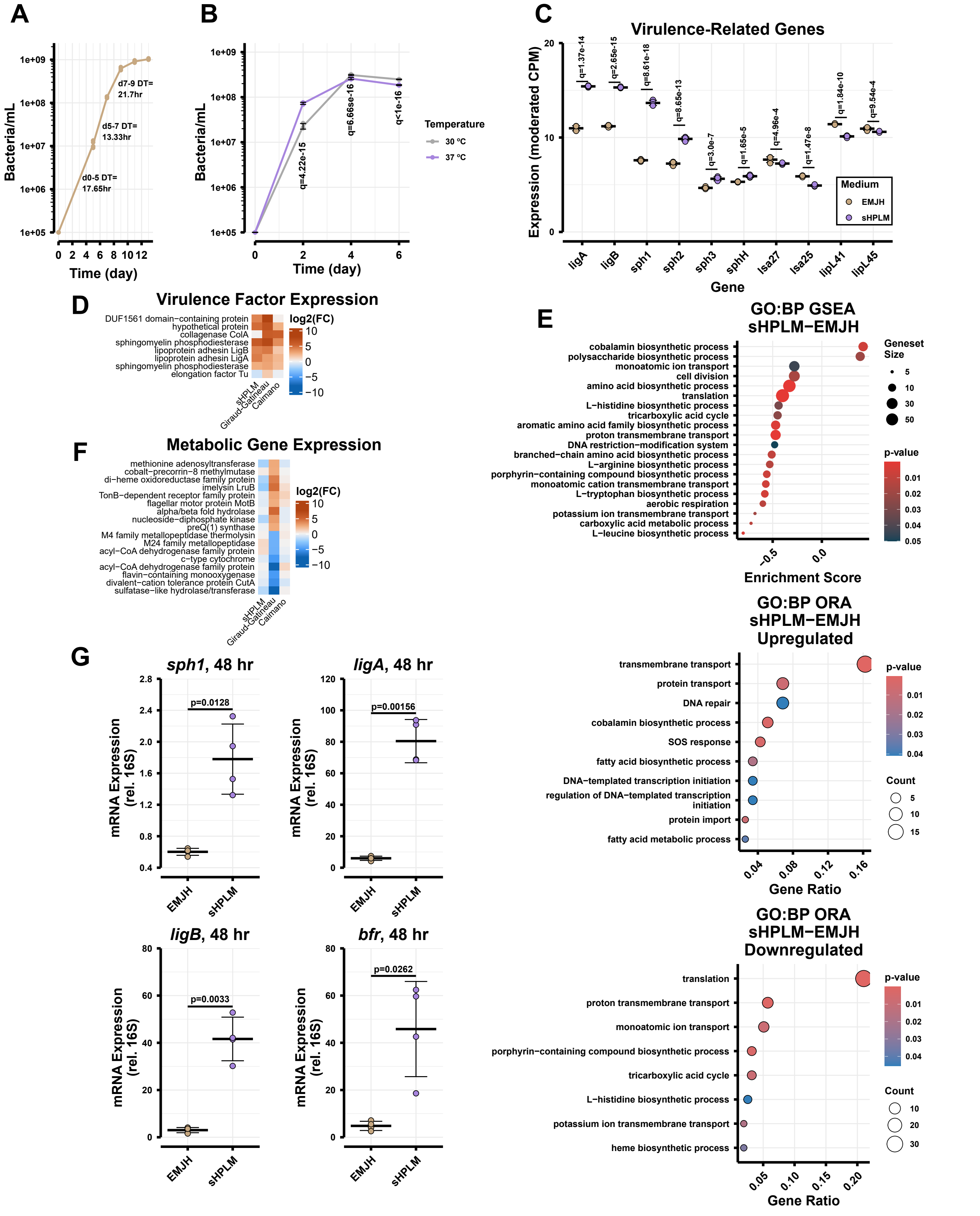
A
Bacteria/mL
Time (day)
d0-5 DT=
17.65hr
d5-7 DT=
13.33hr
d7-9 DT=
21.7hr
B
Bacteria/mL
Time (day)
q=4.22e-15
q=6.66se-16
q<1e-16
Temperature
30 °C
37 °C
C
Virulence-Related Genes
Expression (moderated CPM)
q=1.37e-14
q=2.65e-15
q=8.61e-18
q=8.65e-13
q=3.0e-7
q=1.65e-5
q=4.96e-4
q=1.47e-8
q=1.84e-10
q=9.54e-4
Medium
EMJH
sHPLM
ligA
ligB
sph1
sph2
sph3
sphH
lsa27
lsa25
lipL41
lipL45
Gene
D
Virulence Factor Expression
DUF1561 domain−containing protein
hypothetical protein
collagenase ColA
sphingomyelin phosphodiesterase
lipoprotein adhesin LigB
lipoprotein adhesin LigA
sphingomyelin phosphodiesterase
elongation factor Tu
sHPLM
Giraud-Gatineau
Caimano
log2(FC)
E
GO:BP GSEA
sHPLM−EMJH
cobalamin biosynthetic process
polysaccharide biosynthetic process
monoatomic ion transport
cell division
amino acid biosynthetic process
translation
L−histidine biosynthetic process
tricarboxylic acid cycle
aromatic amino acid family biosynthetic process
proton transmembrane transport
DNA restriction−modification system
branched−chain amino acid biosynthetic process
L−arginine biosynthetic process
porphyrin−containing compound biosynthetic process
monoatomic cation transmembrane transport
L−tryptophan biosynthetic process
aerobic respiration
potassium ion transmembrane transport
carboxylic acid metabolic process
L−leucine biosynthetic process
Geneset Size
p-value
Enrichment Score
F
Metabolic Gene Expression
methionine adenosyltransferase
cobalt−precorrin−8 methylmutase
di−heme oxidoreductase family protein
imelysin LruB
TonB−dependent receptor family protein
flagellar motor protein MotB
alpha/beta fold hydrolase
nucleoside−diphosphate kinase
preQ(1) synthase
M4 family metallopeptidase thermolysin
M24 family metallopeptidase
acyl−CoA dehydrogenase family protein
c−type cytochrome
acyl−CoA dehydrogenase family protein
flavin−containing monooxygenase
divalent−cation tolerance protein CutA
sulfatase−like hydrolase/transferase
sHPLM
Giraud-Gatineau
Caimano
log2(FC)
GO:BP ORA
sHPLM−EMJH
Upregulated
transmembrane transport
protein transport
DNA repair
cobalamin biosynthetic process
SOS response
fatty acid biosynthetic process
DNA−templated transcription initiation
regulation of DNA−templated transcription initiation
protein import
fatty acid metabolic process
p-value
Count
Gene Ratio
G
sph1, 48 hr
p=0.0128
ligA, 48 hr
p=0.00156
ligB, 48 hr
p=0.0033
bfr, 48 hr
p=0.0262
mRNA Expression (rel. 16S)
EMJH
sHPLM
GO:BP ORA
sHPLM−EMJH
Downregulated
translation
proton transmembrane transport
monoatomic ion transport
porphyrin−containing compound biosynthetic process
tricarboxylic acid cycle
L−histidine biosynthetic process
potassium ion transmembrane transport
heme biosynthetic process
p-value
Count
Gene Ratio
