# Supplementary material for: Glutamine from plasma-like medium fuels nitrogen metabolism and influences proliferation in Leptospira interrogans
Source: PLoS Pathog. 2026 Oct 8;22(10):e1014534. doi: 10.1371/journal.ppat.1014534 (PMC13649119; doi:10.1371/journal.ppat.1014534)
Supplement: Supplementary file 2 [file ppat.1014534.s002.pdf]

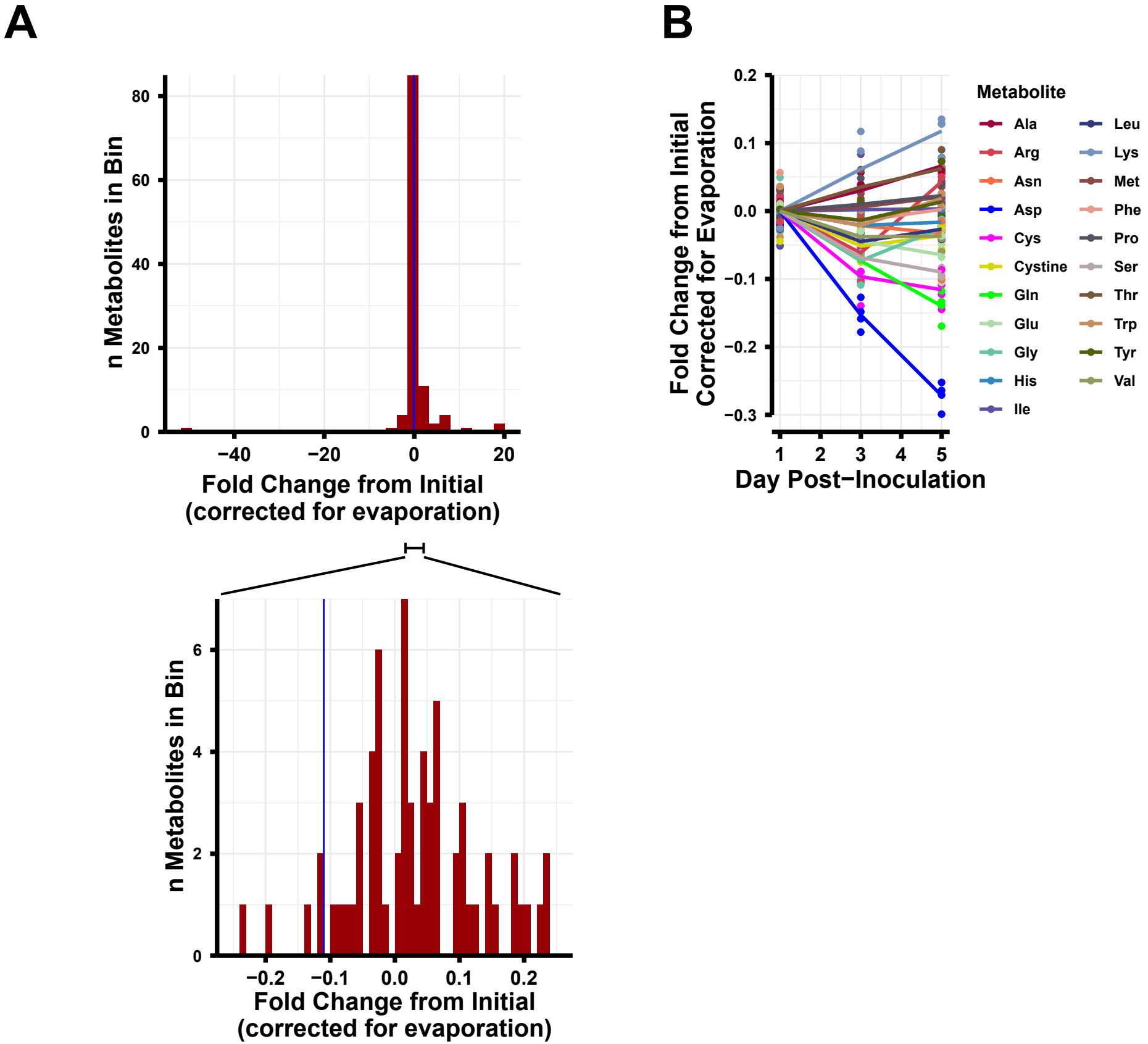
A
n Metabolites in Bin
80
60
40
20
0
−40
−20
0
20
Fold Change from Initial
(corrected for evaporation)
n Metabolites in Bin
6
4
2
0
−0.2
−0.1
0.0
0.1
0.2
Fold Change from Initial
(corrected for evaporation)
B
Fold Change from Initial
Corrected for Evaporation
0.2
0.1
0.0
−0.1
−0.2
−0.3
1
2
3
4
5
Day Post−Inoculation
Metabolite
Ala
Arg
Asn
Asp
Cys
Cystine
Gln
Glu
Gly
His
Ile
Leu
Lys
Met
Phe
Pro
Ser
Thr
Trp
Tyr
Val
