# Supplementary material for: Glutamine from plasma-like medium fuels nitrogen metabolism and influences proliferation in Leptospira interrogans
Source: PLoS Pathog. 2026 Oct 8;22(10):e1014534. doi: 10.1371/journal.ppat.1014534 (PMC13649119; doi:10.1371/journal.ppat.1014534)
Supplement: Supplementary file 3 [file ppat.1014534.s003.pdf]

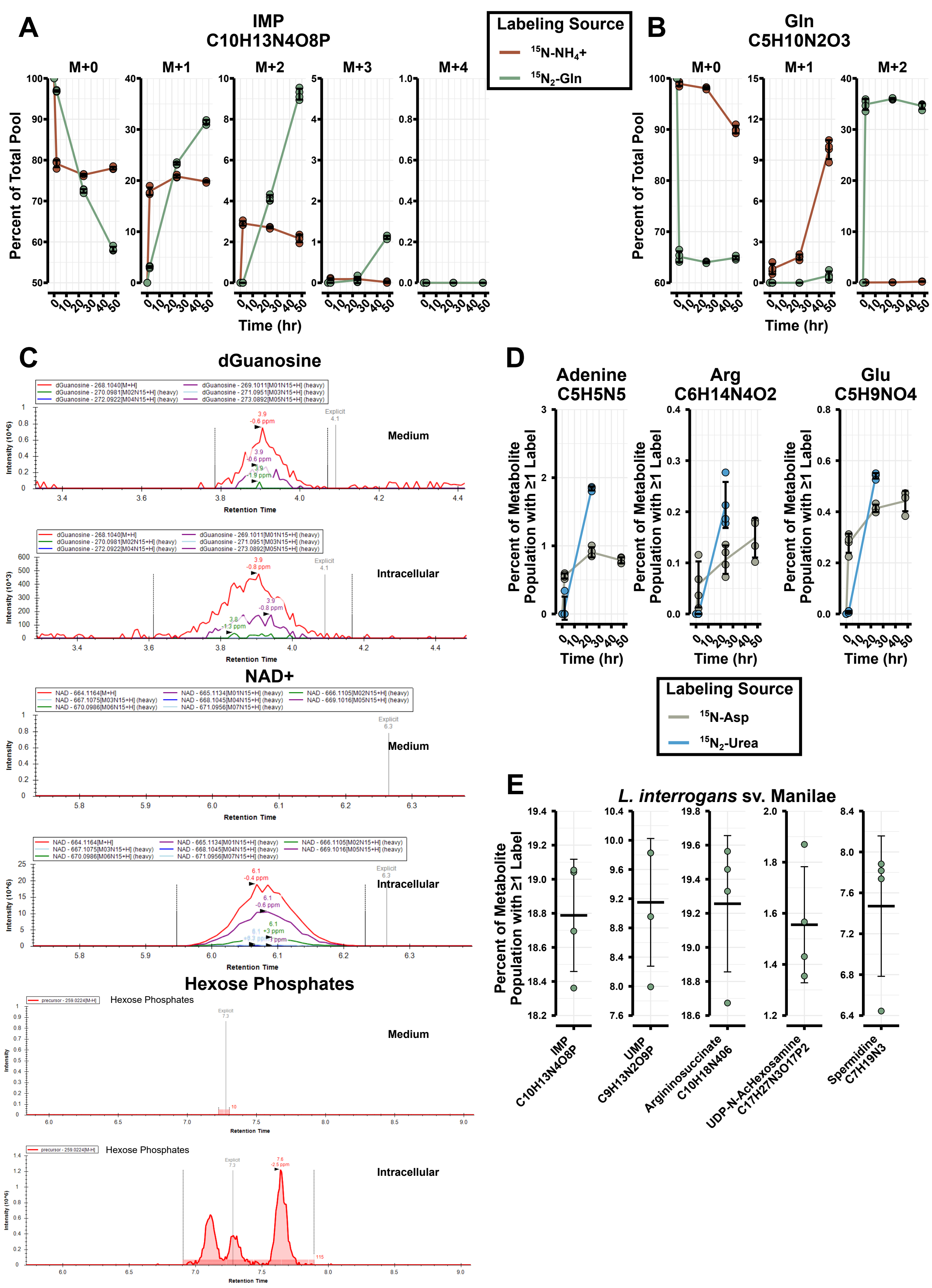

A
IMP C10H13N4O8P
M+0
M+1
M+2
M+3
M+4
Percent of Total Pool
Time (hr)
Labeling Source
15N-NH4+
15N2-Gln
B
Gln C5H10N2O3
C
dGuanosine
Medium
Intracellular
Retention Time
NAD+
Hexose Phosphates
D
Adenine C5H5N5
Arg C6H14N4O2
Glu C5H9NO4
Percent of Metabolite Population with ≥1 Label
15N-Asp
15N2-Urea
E
L. interrogans sv. Manilae
IMP C10H13N4O8P
UMP C9H13N2O9P
Argininosuccinate C10H18N4O6
UDP-N-AcHexosamine C17H27N3O17P2
Spermidine C7H19N3
