# Supplementary material for: Glutamine from plasma-like medium fuels nitrogen metabolism and influences proliferation in Leptospira interrogans
Source: PLoS Pathog. 2026 Oct 8;22(10):e1014534. doi: 10.1371/journal.ppat.1014534 (PMC13649119; doi:10.1371/journal.ppat.1014534)
Supplement: Supplementary file 4 [file ppat.1014534.s004.pdf]

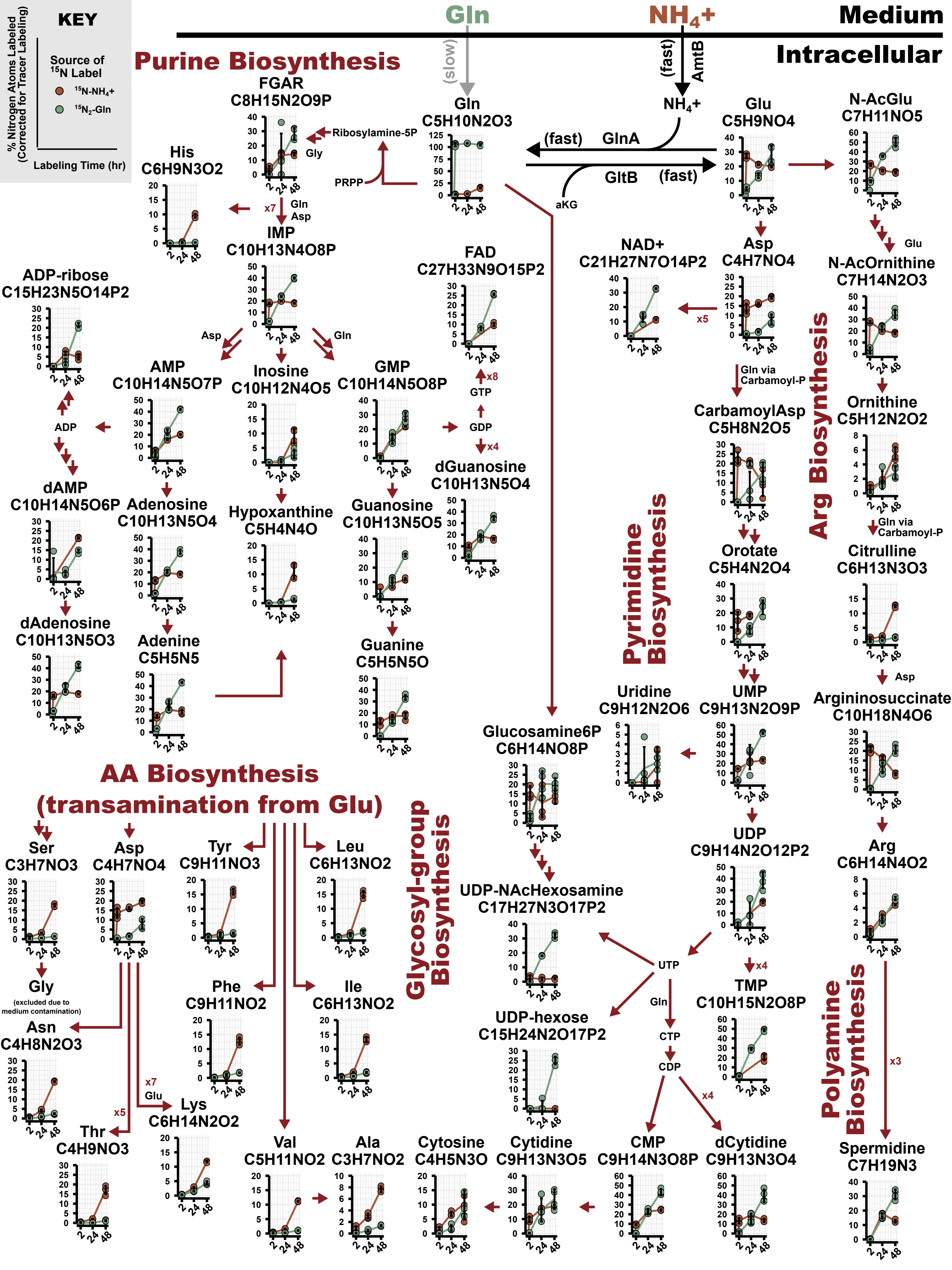

KEY
% Nitrogen Atoms Labeled (Corrected for Tracer Labeling)
Source of 15N Label
15N-NH4+
15N2-Gln
Labeling Time (hr)
Gln
NH4+
Medium
Intracellular
(slow)
(fast)
AmtB
Purine Biosynthesis
FGAR C8H15N2O9P
Ribosylamine-5P
Gly
PRPP
His C6H9N3O2
x7
Gln
Asp
IMP C10H13N4O8P
Gln C5H10N2O3
NH4+
(fast)
GlnA
GltB
(fast)
aKG
Glu C5H9NO4
N-AcGlu C7H11NO5
FAD C27H33N9O15P2
NAD+ C21H27N7O14P2
x5
Asp C4H7NO4
Glu
N-AcOrnithine C7H14N2O3
ADP-ribose C15H23N5O14P2
Asp
Gln
AMP C10H14N5O7P
Inosine C10H12N4O5
GMP C10H14N5O8P
x8
GTP
GDP
x4
ADP
dGuanosine C10H13N5O4
Gln via Carbamoyl-P
CarbamoylAsp C5H8N2O5
Arg Biosynthesis
Ornithine C5H12N2O2
Gln via Carbamoyl-P
dAMP C10H14N5O6P
Adenosine C10H13N5O4
Hypoxanthine C5H4N4O
Guanosine C10H13N5O5
Orotate C5H4N2O4
Citrulline C6H13N3O3
Pyrimidine Biosynthesis
dAdenosine C10H13N5O3
Adenine C5H5N5
Guanine C5H5N5O
Asp
Uridine C9H12N2O6
UMP C9H13N2O9P
Argininosuccinate C10H18N4O6
Glucosamine6P C6H14NO8P
AA Biosynthesis (transamination from Glu)
UDP C9H14N2O12P2
Arg C6H14N4O2
Ser C3H7NO3
Asp C4H7NO4
Tyr C9H11NO3
Leu C6H13NO2
Glycosyl-group Biosynthesis
UDP-NAcHexosamine C17H27N3O17P2
Gly
(excluded due to medium contamination)
UTP
x4
TMP C10H15N2O8P
Phe C9H11NO2
Ile C6H13NO2
Gln
Asn C4H8N2O3
UDP-hexose C15H24N2O17P2
CTP
CDP
x3
Polyamine Biosynthesis
x7
Glu
x5
Lys C6H14N2O2
x4
Thr C4H9NO3
Val C5H11NO2
Ala C3H7NO2
Cytosine C4H5N3O
Cytidine C9H13N3O5
CMP C9H14N3O8P
dCytidine C9H13N3O4
Spermidine C7H19N3
