# Supplementary material for: Glutamine from plasma-like medium fuels nitrogen metabolism and influences proliferation in Leptospira interrogans
Source: PLoS Pathog. 2026 Oct 8;22(10):e1014534. doi: 10.1371/journal.ppat.1014534 (PMC13649119; doi:10.1371/journal.ppat.1014534)
Supplement: Supplementary file 5 [file ppat.1014534.s005.pdf]

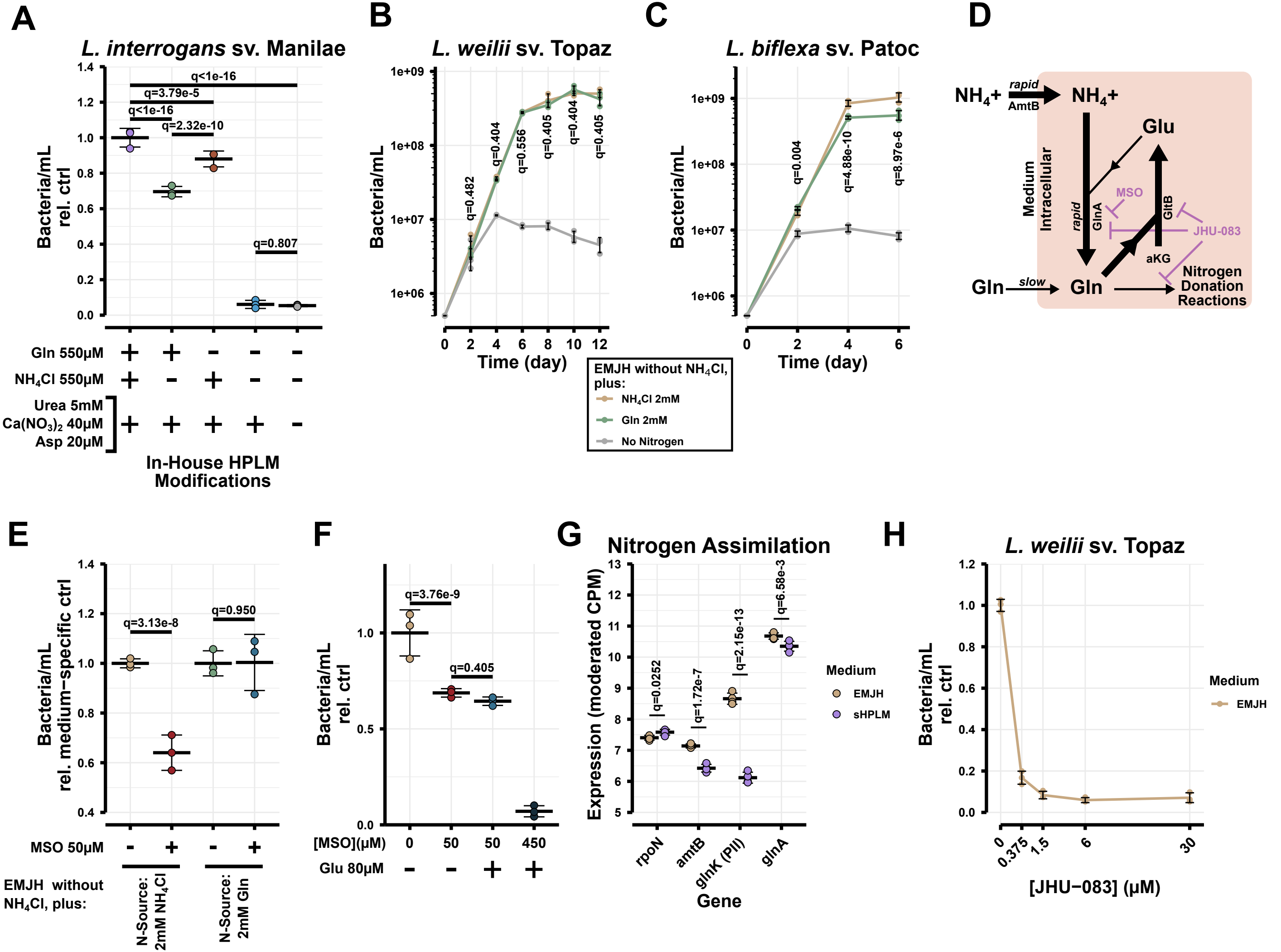
A
L. interrogans sv. Manilae
Bacteria/mL rel. ctrl
q<1e-16
q=3.79e-5
q<1e-16
q=2.32e-10
q=0.807
Gln 550μM + + - - -
NH4Cl 550μM + - + - -
Urea 5mM
Ca(NO3)2 40μM
Asp 20μM
+ + + + -
In-House HPLM Modifications
B
L. weilii sv. Topaz
Bacteria/mL
q=0.482
q=0.404
q=0.556
q=0.405
q=0.404
q=0.405
Time (day)
C
L. biflexa sv. Patoc
Bacteria/mL
q=0.004
q=4.88e-10
q=8.97e-6
Time (day)
EMJH without NH4Cl, plus:
NH4Cl 2mM
Gln 2mM
No Nitrogen
D
NH4+
rapid
AmtB
NH4+
Glu
Medium
Intracellular
rapid
GlnA
MSO
GltB
JHU-083
aKG
Gln
slow
Gln
Nitrogen Donation Reactions
E
Bacteria/mL rel. medium−specific ctrl
q=3.13e-8
q=0.950
MSO 50μM - + - +
EMJH without NH4Cl, plus:
N-Source: 2mM NH4Cl
N-Source: 2mM Gln
F
Bacteria/mL rel. ctrl
q=3.76e-9
q=0.405
[MSO](μM) 0 50 50 450
Glu 80μM - - + +
G
Nitrogen Assimilation
Expression (moderated CPM)
q=0.0252
q=1.72e-7
q=2.15e-13
q=6.58e-3
rpoN
amtB
glnK (PII)
glnA
Gene
Medium
EMJH
sHPLM
H
L. weilii sv. Topaz
Bacteria/mL rel. ctrl
[JHU−083] (μM)
Medium
EMJH
