# Supplementary material for: Glutamine from plasma-like medium fuels nitrogen metabolism and influences proliferation in Leptospira interrogans
Source: PLoS Pathog. 2026 Oct 8;22(10):e1014534. doi: 10.1371/journal.ppat.1014534 (PMC13649119; doi:10.1371/journal.ppat.1014534)
Supplement: Supplementary file 6 [file ppat.1014534.s006.pdf]

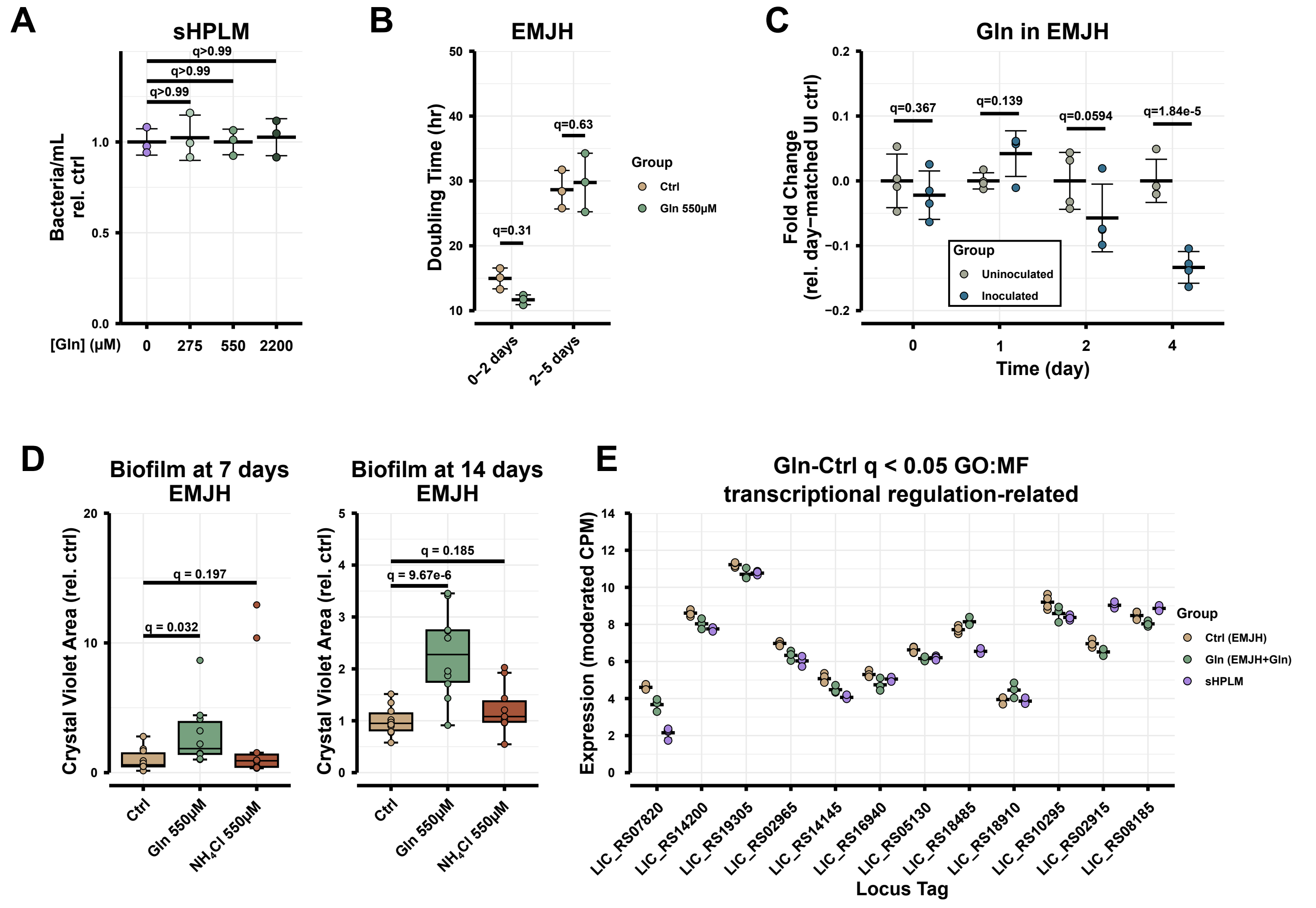

A
sHPLM
q>0.99
q>0.99
q>0.99
Bacteria/mL rel. ctrl
[Gln] (µM)
0
275
550
2200
B
EMJH
Doubling Time (hr)
q=0.31
q=0.63
0–2 days
2–5 days
Group
Ctrl
Gln 550µM
C
Gln in EMJH
Fold Change (rel. day−matched UI ctrl)
q=0.367
q=0.139
q=0.0594
q=1.84e-5
Group
Uninoculated
Inoculated
Time (day)
D
Biofilm at 7 days EMJH
Biofilm at 14 days EMJH
Crystal Violet Area (rel. ctrl)
q = 0.197
q = 0.032
q = 0.185
q = 9.67e-6
Ctrl
Gln 550µM
NH4Cl 550µM
E
Gln-Ctrl q < 0.05 GO:MF transcriptional regulation-related
Expression (moderated CPM)
Group
Ctrl (EMJH)
Gln (EMJH+Gln)
sHPLM
LIC_RS07820
LIC_RS14200
LIC_RS19305
LIC_RS02965
LIC_RS14145
LIC_RS16940
LIC_RS05130
LIC_RS18485
LIC_RS18910
LIC_RS10295
LIC_RS02915
LIC_RS08185
Locus Tag
